# Fitness Landscape of Antibiotic Tolerance in *Pseudomonas aeruginosa* Biofilms

**DOI:** 10.1371/journal.ppat.1002298

**Published:** 2011-10-20

**Authors:** Sasan Amini, Alison K. Hottes, Lincoln E. Smith, Saeed Tavazoie

**Affiliations:** Department of Molecular Biology & Lewis-Sigler Institute for Integrative Genomics, Princeton University, Princeton, New Jersey, United States of America; University of Washington, United States of America

## Abstract

Bacteria in biofilms have higher antibiotic tolerance than their planktonic counterparts. A major outstanding question is the degree to which the biofilm-specific cellular state and its constituent genetic determinants contribute to this hyper-tolerant phenotype. Here, we used genome-wide functional profiling of a complex, heterogeneous mutant population of *Pseudomonas aeruginosa* MPAO1 in biofilm and planktonic growth conditions with and without tobramycin to systematically quantify the contribution of each locus to antibiotic tolerance under these two states. We identified large sets of mutations that contribute to antibiotic tolerance predominantly in the biofilm or planktonic setting only, offering global insights into the differences and similarities between biofilm and planktonic antibiotic tolerance. Our mixed population-based experimental design recapitulated the complexity of natural biofilms and, unlike previous studies, revealed clinically observed behaviors including the emergence of quorum sensing-deficient mutants. Our study revealed a substantial contribution of the cellular state to the antibiotic tolerance of biofilms, providing a rational foundation for the development of novel therapeutics against *P. aeruginosa* biofilm-associated infections.

## Author Summary

Biofilms, matrix-enclosed surface-colonized communities of bacteria, are extremely resistant to antimicrobial agents, withstanding concentrations of antibiotics orders of magnitude higher compared to free-swimming planktonic cells. This is a well-established characteristic of infections caused by the opportunistic pathogen *Pseudomonas aeruginosa*, the major cause of morbidity in cystic fibrosis patients and a frequent cause of nosocomial infections, and *Pseudomonas* infections generally persist despite the use of long-term antibiotic therapy. Nonetheless, the genetic basis of the hyper-tolerance of biofilms to antimicrobial agents is poorly understood. In this study, we use a genome-wide genetic footprinting technology to systematically quantify the contribution of each locus in *P. aeruginosa* to antibiotic tolerance in both biofilm and planktonic states. Comparing and contrasting the genome-wide genetic profile of these two physiological states revealed that large sets of genes modulate antibiotic tolerance as a function of the cellular state.

## Introduction

Biofilms are ubiquitous in nature, and the majority of human bacterial infections involve biofilms [1], [2]. While biofilms contain cells with a heterogeneous range of states [3], on average, bacteria in biofilms have a much higher—up to 1000-fold— antibiotic tolerance than their planktonic counterparts [4].

A case in point is P. aeruginosa, the major cause of morbidity in cystic fibrosis patients [5] and a frequent cause of nosocomial infections [6]. In the lungs of cystic fibrosis patients, P. aeruginosa persists as a biofilm, which further enhances the organism's inherently high antibiotic tolerance [7]. Aerosolized tobramycin, an aminoglycoside, is commonly prescribed to combat P. aeruginosa infections in cystic fibrosis patients [8]. However, during the course of treatment, the drug's efficacy typically decreases as adaptive mutations accumulate leading to the emergence of hyper-tolerant mutants [9], [10]. In an attempt to combat the problem, aminoglycoside tolerance, and more specifically tobramycin tolerance, has been studied extensively in both the biofilm and planktonic states in P. aeruginosa. A number of factors are thought to be involved including oxidative phosphorylation [11], [12], [13], lipopolysaccharide (LPS) composition [11], cyclic di-guanosine monophosphate (c-di-GMP) levels [14], quorum sensing [15], and membrane permeability [11].

In spite of the vast amount of work on the subject, our understanding of the connection between biofilms and antibiotic tolerance remains incomplete. For example, while bacteria in biofilms are generally known to be more tolerant of antimicrobial agents, it is still not clear if strains that are better at biofilm formation necessarily have higher antibiotic tolerance.

To date, several loci have been linked to aminoglycoside tolerance in Pseudomonas. Some, including ndvB, pvrR, arr, and the PA1874-PA1877 efflux pump genes, modulate aminoglycoside tolerance only in the biofilm state [14], [16], [17], [18] and others such as amgRS, mexXY-oprM, and the pel locus have a general impact on tolerance independent of the cellular state [19], [20], [21], [22]. The identified genes, many of which are strain specific [17], [19], belong to a variety of different biological processes including efflux pumping (PA1874–7 and mexXY-oprM), polysaccharide biosynthesis (pel and ndvB), and signaling (arr, pvrR, amgRS). These examples, however, do not provide a comprehensive perspective of antibiotic tolerance in different cellular states, and the extent to which planktonic and biofilm antibiotic tolerances share similar mechanisms and genetic components has not been systematically explored. Additionally, the most commonly used antibiotic sensitivity assays, which are carried out in monocultures of homogenous mutants, do not capture the complex interactions between mutants and the heterogeneous populations from which they emerge.

In order to address these shortcomings, we designed an experimental approach capable of identifying mutants with enhanced antibiotic tolerance in the context of a diverse population. To this end, we adapted and optimized a transposon mutagenesis and genetic footprinting technology [23] for *P. aeruginosa* and used it to quantify the contribution of each *P. aeruginosa* locus to tobramycin tolerance in the biofilm and planktonic states. Our novel experimental design recapitulated many behaviors observed in clinical isolates, such as the high fitness of quorum sensing-deficient mutants [24].

Our results indicate that large sets of loci contribute to antibiotic tolerance predominantly in the biofilm or planktonic setting only and reveal how the cellular state and multi-cellular interactions combine to impact the response to an antibiotic challenge.

## Results

### Fitness Landscape of Biofilm Formation Capacity and Antibiotic Tolerance

**Figure 1:**
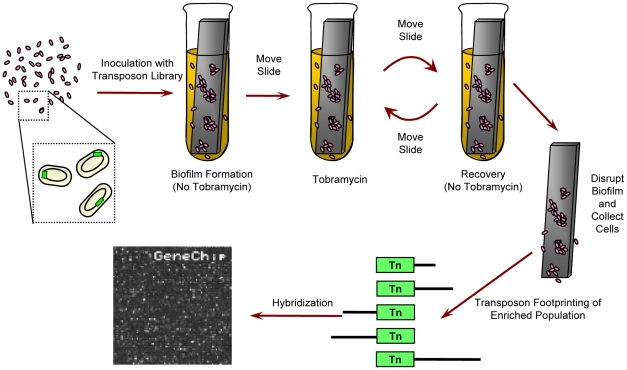
Experimental design. For biofilm experiments, a transposon insertion library was given 24 hours to form a biofilm on a plastic slide in media lacking tobramycin. Next, the slide and the attached biofilm were moved to fresh media with tobramycin for another 24 hours, and then the biofilm was allowed to recover in fresh, drug-free media for an additional 24 hours. After repeating the drug exposure and recovery a second time, the biofilm was disrupted and the cells were collected. Abundance of individual mutants was determined using microarray-based genetic footprinting. Planktonic experiments were similar except the slide was not included and cultures were shaken. Tobramycin was omitted from “no drug” controls. In all cases, containers were sealed. See Materials and Methods for details.

To explore the genetic basis of the emergence of antibiotic tolerant mutants in *P. aeruginosa* biofilms, we allowed a library of transposon insertion mutants to form a biofilm *en masse* and then challenged the population with tobramycin (Figure 1). Then, for each locus, we used the relative abundance of transposon insertions before and after the selection as an indicator of the contribution of that locus to biofilm-mediated tobramycin tolerance. To distinguish loci that modulate antibiotic tolerance specifically in biofilms from those that have planktonic effects, modify biofilm formation capacities, or alter growth rates in the media itself, we performed similar experiments on biofilms in the absence of drug (Bio-ND) and in planktonic cultures with and without tobramycin (Pla-TOB and Pla-ND, respectively). Biofilms of wild-type-cells exposed to the chosen tobramycin concentration (the Bio-TOB condition) had 2% the number of viable cells as untreated biofilms, while planktonic cultures had only 0.02% the number of viable cells of their unexposed counterparts (Figure S1). A comparative analysis of the fitness landscape in the four experimental conditions indicated that large sets of genes contribute to antibiotic tolerance primarily in biofilm or planktonic conditions only. Furthermore, even among the many genes that modulate tobramycin tolerance in both planktonic cultures and biofilms, the relative contribution of individual loci frequently varies as a function of cellular state.

**Figure 2:**
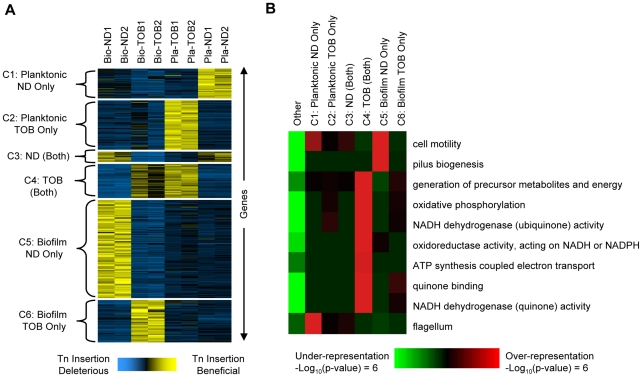
Gene- and pathway-level analysis of fitness profiles. Comparative analysis of genome-wide footprinting data suggests that transposon insertions in or near 586 genes (see Dataset S3 for a complete list of these genes) cause reproducible, condition-dependent behavior that increases fitness in at least one setting (see Protocol S1). (A) The 586 genes (rows) were arranged using K-means clustering into six clusters shown on the left (C1 through C6). The hybridization scores shown for each gene were mean-centered and normalized to a standard deviation of one. This commonly used normalization puts each gene's fitness profile on a similar scale and facilitates comparison between the different conditions. Yellow indicates those conditions where mutants with transposons in or near the indicated gene underwent the greatest increases in abundance. Blue indicates conditions where transposons in or near the same gene were either deleterious or were slightly beneficial and resulted in a comparatively small increase in abundance. Column labels indicate the experimental condition: Bio-ND and Bio-TOB refer to transposon insertion libraries grown as biofilms and treated with no drug or tobramycin, respectively, and Pla-ND and Pla-TOB refer to libraries grown planktonically without or with tobramycin. Two biological replicates were performed in each condition and numbers indicate the repetition number. Gene names and annotations are in Dataset S3. (B) iPAGE was used to look for enrichment and depletion of functional categories (rows) among clusters C1 through C6 plus the set of genes not in any cluster (columns). Red (green) indicates that genes in the cluster were enriched (depleted) for the indicated category.

The level of antibiotic challenge was chosen to be sufficiently severe to enable the identification of clinically relevant pathways that contribute to the emergence of hyper-resistant mutants. Necessarily, this design constraint limited our ability to discover loci in which genetic perturbations increase antibiotic sensitivity and led us to focus on the set of mutants of above-average fitness. Overall, we found that transposon insertions within or in the vicinity of any of 586 open reading frames (ORFs) (Figure 2A; see Dataset S3 for a complete list of these ORFs) cause a reproducible, condition-dependent fitness increase in at least one experimental setting (see Protocol S1).

Most transposon insertions identified here likely cause null alleles, while others possibly act by increasing the expression of neighboring genes [23]. Phenotypes similar to those observed in the identified mutants could arise naturally by similar transposition events or by a range of other alterations including nonsense mutations or frameshift-causing indels. Therefore, the identified mutations have clear implications for the emergence of hyper-tolerant mutants within pathologic biofilms treated with drug. Regardless of the exact mechanism employed, the results indicate that *P. aeruginosa* has a large mutational target for increasing antibiotic tolerance.

In order to identify biological pathways that contribute to the condition-specific fitnesses observed, we first partitioned the 586 genes identified into six clusters (labeled C1 through C6 as shown in Figure 2A) based on their fitness profiles. Next, we used iPAGE [25] to search for functional categories enriched or depleted in each cluster (Figure 2B). Disruptions in many genes whose products function in oxidative phosphorylation were, for example, beneficial in the presence of tobramycin in both the biofilm and planktonic challenges (cluster C4, Figure 2B). The role of the electron transport chain in causing oxidative stress and ultimately death following exposure to lethal concentrations of bactericidal antibiotics was previously described [26], and increased aminoglycoside tolerance resulting from disruption of the pathway components in the planktonic state has been observed in a wide range of species including *P. aeruginosa* [11], [26], [27].

Although disruptions of oxidative phosphorylation components are beneficial in both biofilm and planktonic conditions, unlike the Pla-TOB condition, oxidative phosphorylation mutants do not dominate the population in the Bio-TOB condition. In particular, mutants with transposon insertions in the main NADH dehydrogenase operon (PA2637-PA2649) appear significantly lower in lists of the most abundant insertions (Wilcoxon matched pairs signed-rank test *p*-value  = 0.033) following Bio-TOB selections compared to Pla-TOB selections.

iPAGE did not identify any significant functional enrichments specific to either the Pla-TOB or Bio-TOB challenges (clusters C2 and C6, respectively), likely due to the poor quality of the *P. aeruginosa* genome's annotation. However, we observed functional enrichment/depletion patterns in classes not involving tobramycin. For example, disruptions in genes involved in type-IV pili biosynthesis were beneficial in biofilms in the absence of tobramycin but not in any of the other conditions (cluster C5). The existence of numerous mutants with high fitness in the Bio-ND but not the Bio-TOB selection indicates that the high biofilm-formation capacity is not, in itself, sufficient to increase antibiotic tolerance. Type-IV pili mediate twitching motility [28], and failure of a population to reduce twitching motility results in abnormal biofilm development [29], [30]. While homogeneous cultures of pili mutants are deficient in biofilm formation [31], mixtures of pili mutants and wild-type cells form biofilms with the pili mutants located predominately in the stalks of microcolonies [32].

Additionally, disruption of a different type of motility—flagellum-based swimming—was beneficial specifically in the planktonic enrichments without drug (Figure 2, cluster C1), likely due to the high energetic cost of flagella synthesis and rotation [33]. As cells in biofilms typically do not have flagella [34], and cells lacking flagella are defective in the early stages of biofilm formation [31], the lack of a functional flagella biosynthesis pathway would be expected to be much less beneficial in biofilms.

### Fitness Profiling via Direct Competition Assays

To better understand the contribution of the identified loci to biofilm-mediated tobramycin tolerance, we chose 45 mutants from University of Washington (UW) transposon insertion mutant collection [35] based on the genome-wide footprinting data and individually competed each against a differentially labeled *P. aeruginosa* reference strain in a scaled-down version of the Bio-TOB experiment described above. Mutants chosen belonged mainly to clusters C4 and C6 of Figure 2A (see Protocol S1 and Figure S2 for details). The reference strain served as an internal control for experiment-to-experiment biofilm-formation variability, facilitated between-strain comparisons, and helped mimic natural biofilm conditions where mutants arise in the presence of the parental strain. In order to focus on genes whose role in antibiotic tolerance had not been previously characterized, genes identified in a previous genome-wide study of low-level aminoglycoside resistance [11] were excluded, except for three: *nuoK* (PA2646), *nuoA* (PA2637), and *wzm* (PA5451), which were included as controls.

**Figure 3:**
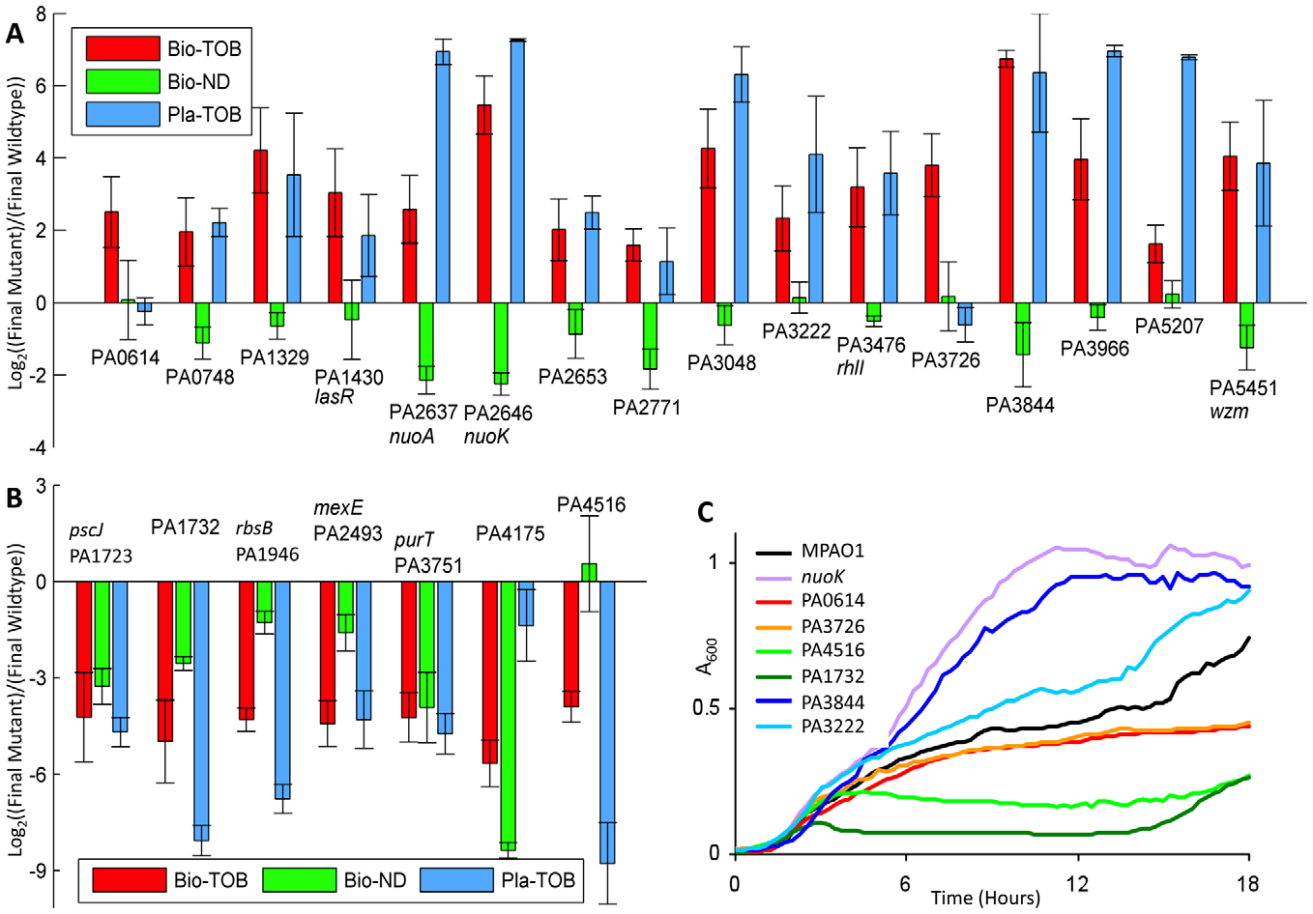
Fitness characterization of candidate mutants in different physiological states. (A, B) Competitions were started with equal amounts of mutant and reference cells. The y-axis indicates the relative count of mutants over the reference strain following one round of the indicated experimental challenge (as explained in Materials and Methods). As cultures undergo different numbers of generations during each type of challenge, values from different challenges for the same mutant are not directly comparable. Error bars indicate the standard error of at least 8, 4, and 3 experiments for the Bio-TOB, Bio-ND, and Pla-ND conditions, respectively. Mutants in (A) have an advantage over wild-type in the Bio-TOB competition; mutants in (B) have a disadvantage. Gene annotations, which were updated from the original genome annotation [48] by BLAST comparisons [14] against the NCBI non-redundant database, are in Table S3. (C) Growth curves for representative strains from panels (A) and (B) with 4 µg/ml tobramycin are shown.

Twenty-three mutants demonstrated fitness changes—16 increases and 7 decreases—in the Bio-TOB challenge beyond that typical of the UW collection (see the ‘Competition Assays' section in the Protocol S1 document), and those mutants were then subjected to similar competitions in Bio-ND and Pla-TOB conditions (Figures 3 and S3). Our inability to replicate the original library observations using the other 22 UW strains is likely due to differences in transposon location and orientation between the UW collection and the original transposon insertion library. Spontaneous mutations in individual strains of the UW collection as well as differences between the parental strains of the original library and the UW collection, which are both MPAO1, might also have contributed.

Among the sixteen mutants with substantially above-average fitness in the Bio-TOB setting, the tobramycin resistance of two strains, the PA3726 and PA0614 mutants, is specific to the biofilm state. Neither performed markedly above-average in the Bio-ND competitions; further analyses of these mutants are presented later. The other 14 mutants displayed at least a mild to moderate tobramycin tolerance in the planktonic state, suggesting that similar pathways confer antibiotic tolerance in both the biofilm and planktonic states (Figure 3A). As cultures in the planktonic condition undergo different numbers of generations compared to the biofilm setting, competition values from Bio-TOB and Pla-TOB challenges for the same mutant are not directly comparable.

Interestingly, both *nuoA* and *nuoK* strains, which are among the fittest in the Pla-TOB condition, have below-average fitness in the Bio-ND condition. Thus, the relative fitness of *nuoA* and *nuoK* mutants in the Bio-TOB setting in competition with a wild-type strain is the result of a combination of two counteracting factors: a general deficiency in biofilm formation and a survival advantage upon exposure to tobramycin. Thus, the biofilm-formation defect could explain why strains defective in oxidative phosphorylation were enriched less strongly in the Bio-TOB library experiments than in the Pla-TOB library experiments. Alternatively, although both the planktonic and biofilm cultures were in sealed containers, increased oxygen availability and usage in the planktonic cultures may have decreased the comparative fitness of the wild-type strain in Pla-TOB conditions [26].

Seven mutants demonstrated a fitness defect in the Bio-TOB competitions (Figure 3B), suggesting that the corresponding transposon insertions enriched in the original library caused over-expression, rather than deletion, phenotypes. Consistent with this hypothesis, the group includes PA2493 (*mexE*, multidrug efflux membrane fusion protein [36]) whose over-expression is known to cause a multi-drug resistance phenotype [37].

### Increased Biofilm Tobramycin Tolerance Is Not Associated with Slow Growth in the Planktonic State

Since fast growing bacteria are more susceptible to antibiotics [38], we generated growth curves in the absence of drug for all 23 strains to determine if an inherently slow growth rate could account for the observed antibiotic tolerance. Figure S4 shows that during the first twelve hours of growth without tobramycin, all of the strains display growth patterns similar to the reference strain, suggesting that exponential phase growth differences are not a major contributor to the observed antibiotic tolerance changes.

To further characterize the antibiotic susceptibility of the strains in the planktonic state, we also generated growth curves for the mutants with 4 and 8 µg/ml tobramycin. With 4 µg/ml tobramycin, half the concentration typically used in this work, the growth rates observed were generally consistent with the fitness measured in the Pla-TOB competitions. Notably, the two mutants (PA3726, and PA0614) that out-competed the reference strain in the Bio-TOB, but not the Pla-TOB competitions, do not exhibit any detectible planktonic growth rate advantage in 4 µg/ml of tobramycin (Figures 3C and S5), which provides complementary evidence that these mutants have average planktonic tobramycin tolerance. With 8 µg/ml tobramycin, the density of most cultures plateaued at less than 20% of the density obtained without drug (Figure S6). The growth curves indicate that the majority of the mutants selected for characterization have a moderate growth advantage over the reference strain in Pla-TOB conditions. As shown in Figure S6, the advantage, however, is far less pronounced than that exhibited by many well-characterized antibiotic-tolerant mutants, such as *nuoA* and *nuoK* strains (NADH dehydrogenase I mutants). While our genome-wide screen identified many loci previously known to cause strong antibiotic tolerance [11], they were largely excluded from the final set in order to focus on genes whose role in antibiotic tolerance had not been previously characterized.

The growth kinetics of the strains with 4 µg/ml and 8 µg/ml of tobramycin (Figures S5 and S6) suggests that the minimum inhibitory concentration (MIC) of most of the mutants analyzed is within 2-fold of that of the wild-type strain. Disk susceptibility assays conducted on a subset of the mutants (PA0748, *nuoK*, PA2771, PA4516, PA2653, and PA3966) yielded zones of inhibition indistinguishable from the reference strain (data not shown). Kill curves for monocultures of the same set of mutants in biofilm and planktonic settings (Figure S7) indicate that 4 of the 6 strains behave similarly to the reference strain in biofilms and are slightly more resistant in planktonic cultures; the *nuoK* strain is less susceptible in both conditions while the PA4516 mutant is more susceptible. The substantial behavioral differences between competition and traditional homogeneous culture assays, combined with the former's similarity to clinical settings, argue that the field should strive to incorporate mixed-population tests into the standard battery. Furthermore, as many of the mutations analyzed likely affect different pathways, strains that accumulate multiple mutations of small effect may exhibit clinically relevant levels of resistance [27], [39].

### Assigning Individual Genes to Biological Processes and Pathways

To better understand the biological pathways and processes that contribute to enhanced biofilm tolerance to antibiotics, we used a combination of experimental and computational approaches to classify the candidate loci into functional classes. The altered tobramycin susceptibility of nine of the 23 mutants analyzed is likely due to modulation of one of three processes previously implicated in antibiotic tolerance: quorum-sensing, oxidative phosphorylation, and membrane permeability (Table S3). The other mutants likely modify pathways not examined here, such as LPS composition [11], or employ novel mechanisms of action.

#### Oxidative phosphorylation

**Figure 4:**
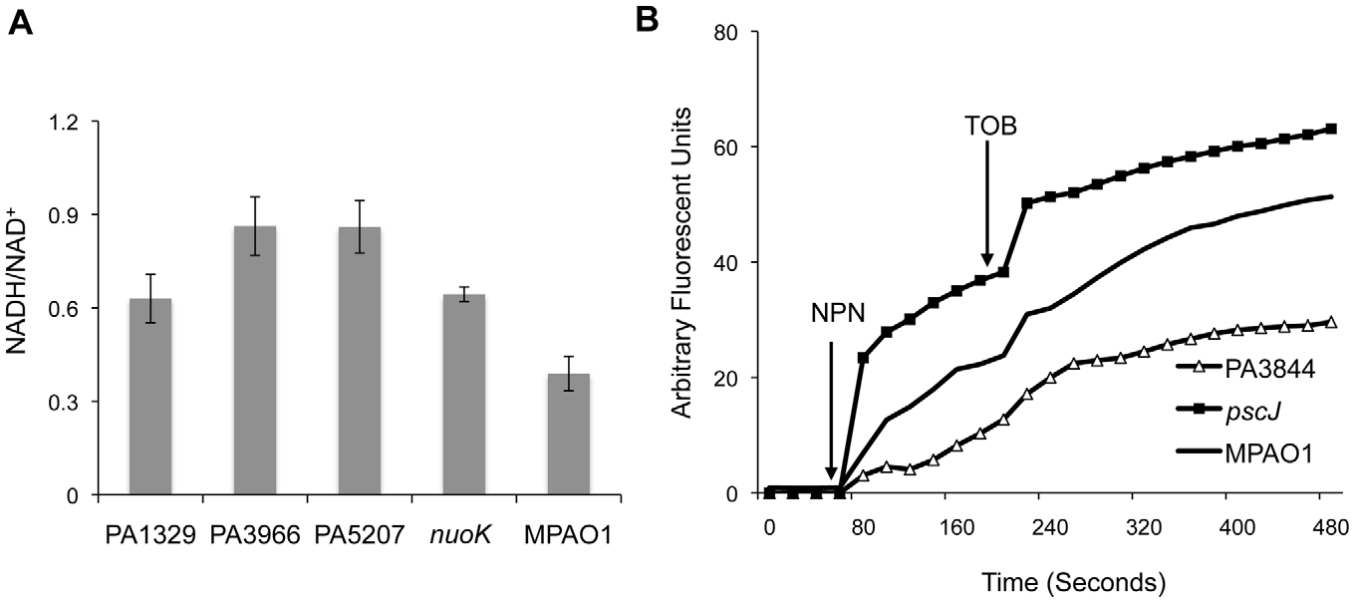
Functional classification of genes associated with antibiotic tolerance in biofilms. (A) The NADH/NAD^+^ ratio was measured for a subset of mutants with the most pronounced growth advantage in the planktonic state with tobramycin. The NADH/NAD+ ratios for the strains shown are significantly higher than wild-type (Student's one-sided t-test p-values: 0.02, 0.002, 0.001, and 0.015 for mutants in PA1329, PA3966, PA5207, and *nuoK*, respectively). Error bars represent the standard error of at least three replicates. (B) The disruptive effect of tobramycin on the outer membrane of different mutants was measured using an NPN assay. NPN and tobramycin were added at the indicated times.

One of the first symptoms of a lethal dose of bactericidal antibiotics is increased oxidation of NADH through the electron transport chain [26], and *P. aeruginosa* strains with defects in energy metabolism, the NADH dehydrogenase complex, or cytochromes are more tolerant of tobramycin [11]. Therefore, we wanted to determine if any of the mutants identified have abnormal NADH/NAD^+^ ratios. As NADH dehydrogenase mutants are particularly fit in Pla-TOB challenges, we focused on the mutants with the most pronounced growth advantage with tobramycin in the planktonic state. In addition to the positive control, *nuoK*, three mutants (PA1329, PA3966, and PA5207) have an elevated NADH/NAD^+^ ratio compared to the wild-type strain (Figures 4A and S8), which could explain why these mutants are more tolerant of tobramycin in both the planktonic and biofilm states.

#### Membrane permeability

To investigate whether any of the candidate mutants have abnormal membrane permeability or increased susceptibility to the disruptive activity of aminoglycosides, we used a 1-N-phenylnaphtylamine (NPN) assay [11], [40]. NPN is a fluorescent probe that has weak fluorescence activity in aqueous solutions but fluoresces substantially in non-polar or hydrophobic environments such as membranes. In the absence of membrane-disruptive stress, NPN has limited access to the outer membrane and shows minimal activity. The introduction of tobramycin, however, compromises outer membrane integrity, allowing more NPN to leak into the interior section of the membrane, leading to increased fluorescence.

The NPN assay indicates that PA1723 (*pscJ*, type III export protein mutant) has a compromised membrane and that the membrane of PA3844 is unusually impermeable (Figure 4B). The behavior of the remaining mutants tested was indistinguishable from the wild-type (Figure S9). This could explain why PA1723 mutants have a relative fitness disadvantage in the presence of tobramycin while PA3844 mutants have an advantage.

#### Quorum sensing

Although others have observed quorum sensing-deficient *P. aeruginosa* to be more sensitive to various antimicrobial agents, including tobramycin [15], in this work, two quorum sensing-deficient mutants, *lasR* (PA1430), which encodes a transcriptional regulator, and *rhlI* (PA3476), which encodes an auto-inducer synthesis protein, displayed fitness advantages in biofilms exposed to tobramycin. This apparent discrepancy may be explained by the ability of some quorum sensing mutants to act as cheaters in mixed populations, allowing the mutants to reap some of the population benefits of quorum sensing without sharing the metabolic burden of activating quorum-sensing downstream processes (see [41] for an example).

A similar situation involving indole, a different signaling molecule, was recently reported [42]. In that case, a small number of resistant mutants produced indole at a fitness cost to themselves. The more sensitive members of the population then sensed the indole and increased production of efflux pumps and oxidative-stress protections, increasing their resistance beyond that exhibited in a homogeneous population.

To investigate whether any of our other mutants of interest perturb the quorum sensing circuitry, we transferred reporter plasmids for each of the two quorum sensing systems to all mutants except those whose annotations suggested a low likelihood of involvement in quorum sensing. As shown in Figures S10 and S11, in addition to the *lasR* and *rhlI* strains, only one strain, the PA1732 mutant, which has low activity in both the *rhl* and *las* systems, was distinguishable from wild-type. Unlike the *lasR* and *rhlI* strains, however, the PA1732 mutant performs poorly in Bio-TOB competitions, possibly due to pleiotrophic effects from disrupting PA1732, which encodes a transglutaminase-like domain containing protein.

#### Microarray expression meta-analysis

To determine if subsets of the genes of interest are co-expressed, and hence more likely to function together, we did a meta-analysis of 255 published expression arrays (See Materials and Methods). The available data, which consist of the response to a variety of stresses and growth conditions in several different genetic backgrounds, contain three main expression classes (Figure S12). The first class contains 2701 genes involved in core metabolic processes including translation, lipid A and nucleotide synthesis, tRNA and rRNA processing, and DNA replication (Figure S13). The second class consists of 2527 genes enriched in type II secretion, cytochrome c oxidase activity, and periplasmic processes. The remaining 320 genes have expression patterns related to each other only weakly. Of the 23 genes of interest, 8 are expressed with the first class, 12 with the second, and 3 with the third, which is consistent with the distribution expected by chance (Figure S14). This indicates that the genes whose disruption affects fitness in biofilms in the presence of tobramycin are not all co-regulated.

Additionally, we used iPAGE to identify functional categories significantly correlated or anti-correlated with each gene of interest at the transcriptional level. While the three large transcriptional classes shown in Figure S12 explain most of the observed patterns, some additional gene-specific patterns appeared. Of particular interest is the correlation of PA2771 (diguanylate-cyclase with GAF domain) expression with genes involved in drug response (p-value  = 1×10^−5^) (Figure S15). It is generally believed that high levels of c-di-GMP increase extracellular matrix formation [43], and c-di-GMP helps induce biofilm growth in response to low levels of tobramycin [14]. Based on this generic model, disruption of PA2771 would be expected to be deleterious in the Bio-ND setting, which agrees with the competition data (Figure 3A).

### Functional Characterization of Loci with Biofilm-Exclusive Tobramycin Fitness Advantages

The PA0614 mutant is one of two strains with a fitness advantage exclusive to the drug-exposed biofilm state. PA0614 is up-regulated by ciprofloxacin challenge [44], has a hydrophobicity profile similar to holins [44], and, as judged by BLAST e-values, is homologous to holins from other *Pseudomonas* species.

**Figure 5:**
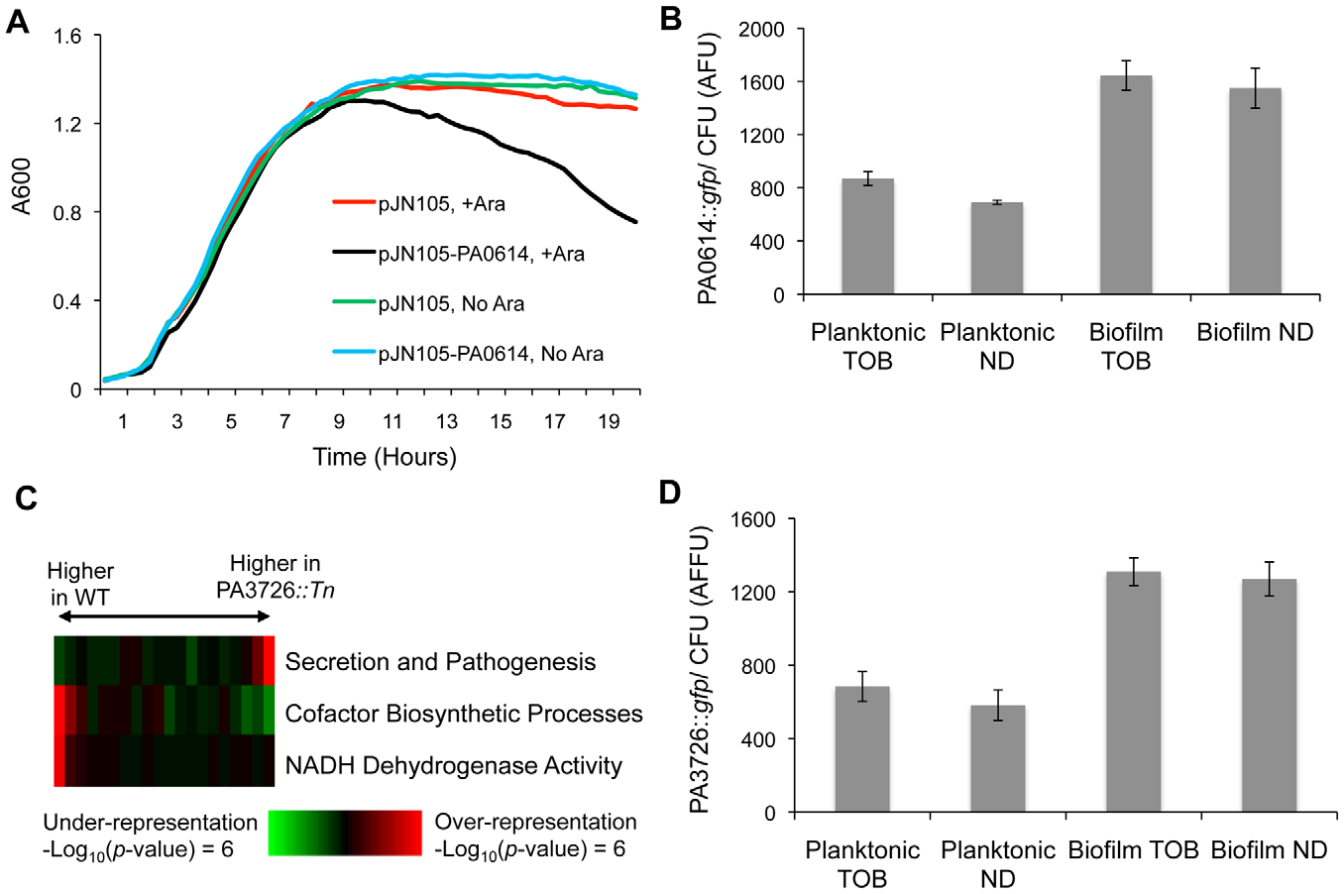
Characterization of loci conferring biofilm-specific tobramycin tolerance. (A) The MPAO1 strain with either an empty vector or a plasmid containing PA0614 under the control of an arabinose-inducible promoter was grown in M63 medium in the presence and absence of 0.2% arabinose (Ara). (B) A *gfp* promoter fusion was used to measure the expression of PA0614 in both biofilm and planktonic settings, in the presence and absence of 8 µg/ml tobramycin. Promoter activities are normalized by colony forming units (CFU). (C) Expression differences between exponentially growing cultures of wild-type and PA3726 mutant cells were sorted and partitioned into 20 equally populated bins, which were subjected to iPAGE analysis. The most informative functional categories are shown. (D) PA3726 promoter activity was measured as described in (B).

To establish that PA0614 is involved in cell lysis, as would be expected for a holin, the PA0614 ORF was cloned downstream of an arabinose-inducible promoter on the low copy number vector pJN105 [45]. Over-expressing PA0614 by adding arabinose to the growth medium lead to increased cell lysis (Figure 5A).

To characterize the role of PA0614 in biofilms, we made a transcriptional fusion of the PA0614-upstream-region to *gfp*. As a control, we first determined that the promoter fusion is, as expected, ciprofloxacin-inducible (Figure S16). Next, we measured PA0614 promoter activity in biofilms and planktonic cells and found that the promoter is almost twice as active in biofilms as in planktonic cells, independent of the presence or absence of tobramycin (Figure 5B). In contrast, the promoter of a random gene, PA3057, did not show state-dependent expression when subjected to the same assay (Figure S17). Thus, the PA0614 gene's increased transcription in biofilms combined with the product's lethal activity, which may synergize with other stresses such as tobramycin treatment, could account for why PA0614 mutants have a competitive advantage specifically in biofilms challenged with tobramycin.

The second mutant we found to have a biofilm-specific tobramycin tolerance has a transposon insertion in PA3726, which encodes a hypothetical protein homologous to the *Salmonella typhimurium* protein YaeQ. In *Escherichia coli* and *S. typhimurium*, YaeQ has been reported to be a suppressor of mutations in *rfaH*, an anti-terminator required for full-length expression of some virulence factor operons [46], although those findings are not without controversy [47].

In order to better understand the biological function of PA3726, we examined the exponential-phase, planktonic mRNA expression of a PA3726 mutant. As shown in Figure 5C, we found that the PA3726 mutant has decreased expression of genes encoding NADH dehydrogenase activity (e.g., *nuoI*, *nuoF*, and *nuoM*) and cofactor biosynthetic processes (e.g., *cobU*, *cobB*, and *cbiD*, which are involved in cobalamin synthesis) and increased expression of secretion and pathogenesis genes (e.g., *exoY*, *pscU*, and *exsC*).

Using a *gfp* transcriptional-fusion reporter construct, we found that PA3726, similar to PA0614, is transcribed more actively in biofilms than planktonic cultures, independent of the presence or absence of tobramycin (Figure 5D). Since PA3726 is expressed more highly in biofilms than planktonic cells, disruption of PA3726 likely has a larger effect in the biofilm state. As PA3726 disruption reduces expression of genes encoding NADH dehydrogenase activity (Figure 5C), the relative decrease in oxidative phosphorylation and increase in tobramycin resistance is likely larger in the biofilm state. Thus, taken together, our results suggest that disrupting PA3726 could reduce NADH dehydrogenase expression preferentially in biofilms, conferring biofilm-specific tobramycin tolerance.

## Discussion

To better understand the role of cellular state and gene-environment interactions in antibiotic tolerance, we examined the relative importance of each gene in *P. aeruginosa* to fitness in the presence of tobramycin as a function of whether the bacterium is living in a biofilm or growing planktonically. Several previous studies identified a small number of *P. aeruginosa* genes whose contribution to antibiotic tolerance depends on whether the cells are in a biofilm or planktonic state [16], [17], [18], but this work represents a substantially more comprehensive and systematic examination of the question. Here, we competed the mutants in a transposon library *en masse* in each of four conditions: biofilms with and without tobramycin, and planktonic growth with and without tobramycin. We then characterized the changes in the population by genetic-footprinting and microarray hybridization.

All the biofilms were formed on plastic slides under static conditions with limited oxygen availability, likely creating micro-aerobic conditions. Cellular physiology in oxygen-limited biofilms is clinically relevant as during chronic, late-stage cystic fibrosis, *P. aeruginosa* grows under reduced oxygen tension and is capable of respiring anaerobically within the thickened airway mucus [48].

Although it is well-known that the biofilm state increases drug tolerance [4], we find that the strains that are most fit in biofilm environments do not necessarily have higher antibiotic tolerance. In fact, our population-level data shows that the set of mutants with high fitness in biofilms (not exposed to tobramycin) has minimal overlap with the set of mutants with high fitness in biofilms in the presence of tobramycin. This implies that the general resistance provided by a biofilm against antibiotics does not protect all members equally and that genetic factors contribute to fitness in the biofilm context.

Moreover, while there is a considerable overlap between the loci that modulate antibiotic tolerance in biofilm and planktonic cells, the relative importance of most genes is state-specific. For example, the vast majority of mutants with the strongest fitness advantages in the biofilm state also have only a weak to moderate advantage in the planktonic state. Such strains, however, perform poorly in planktonic tobramycin challenges when in complex mutant populations due to the presence of a myriad of other strains, such as NADH dehydrogenase mutants, with more pronounced planktonic drug tolerance capacities. Similarly, while NADH dehydrogenase mutants are among the fittest strains in planktonic tobramycin challenges, these mutants exhibit only a comparatively moderate advantage in biofilms exposed to tobramycin. We expect that such differential fitness reflects not only the physiological state of the cells but also environmental differences, such as lower oxygen availability.

**Figure 6:**
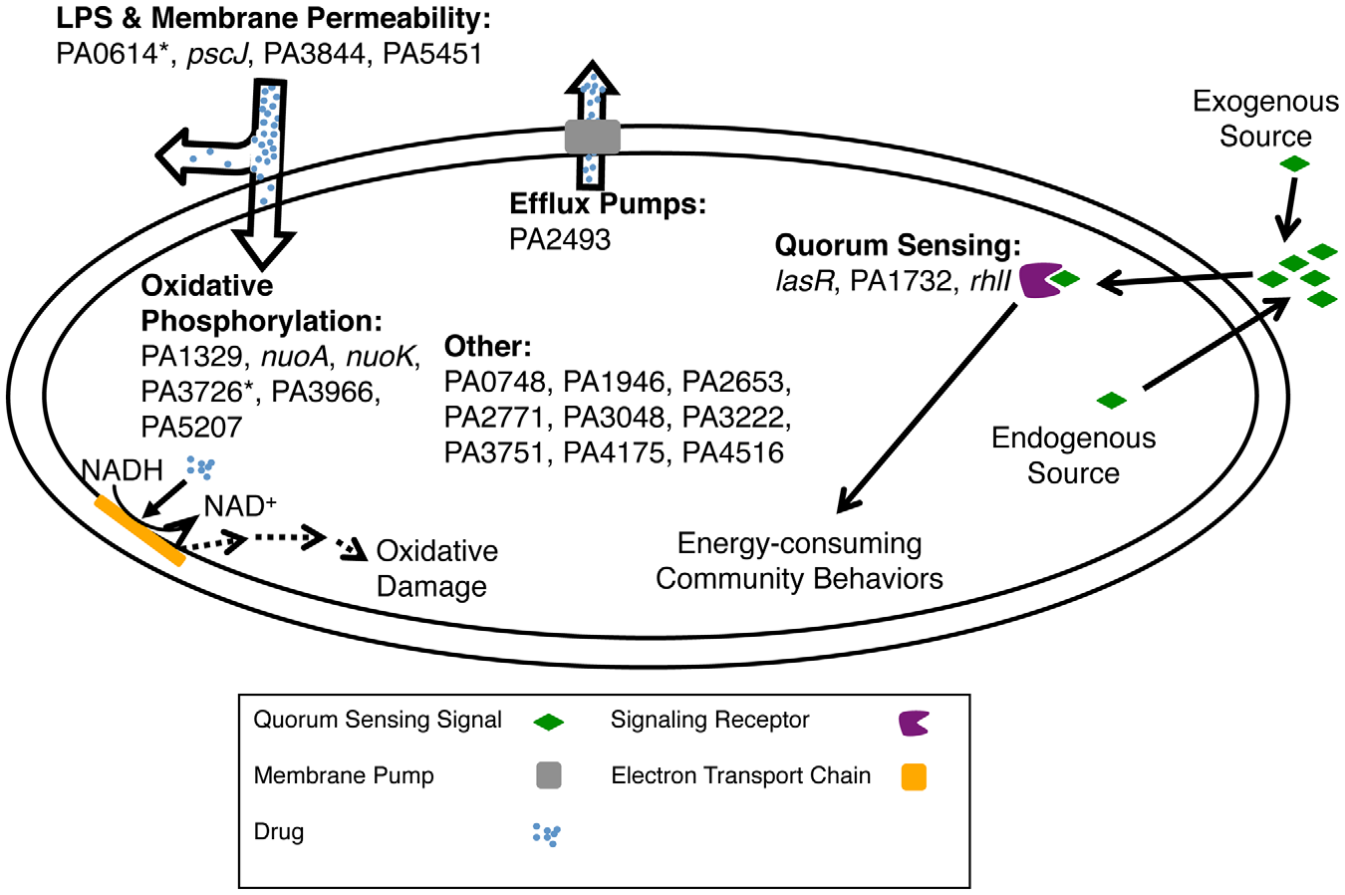
Mechanisms for altering biofilm-mediated antibiotic tolerance. Shown are pathways that the genes from Figure 3A and 3B likely use to modulate biofilm-mediated antibiotic tolerance in *P. aeruginosa*. The “*” indicates that the altered antibiotic-susceptibility is specific to the biofilm state. Genes in the ‘Other' category likely affect uncharacterized pathways or pathways not assayed in this work.

To better understand the pathways contributing to tobramycin tolerance in biofilms, we undertook a broad characterization of a subset of the strains that demonstrated a fitness advantage in biofilms in the presence of tobramycin and whose role in antibiotic tolerance had not been previously identified. We found mutants with changes in membrane permeability, quorum sensing, efflux pump abundance, and oxidative respiration activity—changes previously associated with planktonic antibiotic tolerance [11], [12], [13], [15]. Some mutants, however, did not show changes in any of the above pathways, suggesting that additional mechanisms are at play (Figure 6). One such mechanism that we did not explore is conversion to the RSCV (Rough Small-Colony Variant) state, which is associated with hyper-adherence to solid surfaces and higher antibiotic tolerance [17], [49].

As *P. aeruginosa* cells in microcolonies have elevated mutation rates [50] and many clinical isolates of *P. aeruginosa* are hypermutable [51], each population explores a large part of the fitness landscape. In the course of this real-time evolution, each new mutant starts as a minority and competes against the pre-existing population. In this study, we attempted to capture some elements of natural conditions by analyzing our library of mutants as a heterogeneous pool rather than as homogeneous cultures of individual mutants as has been done more commonly [11], [16]. Each individual mutant was present at low abundance and was tested for a fitness advantage or disadvantage within a diverse population that was expected to function collectively as a wild-type proxy.

Our choice of experimental paradigm leads to some important discrepancies with previous works. For example, while Bjarnsholt *et al.* showed that quorum sensing enhances tobramycin tolerance in *P. aeruginosa* [15], our results indicate that some quorum sensing-defective mutants have a fitness advantage in the presence of tobramycin in competition with a quorum sensing-capable strain. The cheating behavior of quorum-sensing mutants in a mixed population [41] can explain the incongruity, and, indeed, quorum sensing-deficient mutants, specifically *lasR* mutants, have been frequently isolated from *Pseudomonas–*associated infections [24]. Hence, the mixed population approach utilized here, in both the initial selections and the follow-up competitions, appears to capture some real-world, biological phenomena not observed in homogeneous cultures.

Efforts to develop effective therapeutic strategies against *P. aeruginosa* infections can benefit from a thorough understanding of how each gene contributes to the organism's antibiotic tolerance in the range of microenvironments present within an infection. We hope that this work and future studies using similar tools in other natural and clinical isolates will contribute to that effort.

## Methods

### Strains, Media, and Growth Curves

M63 media (100 mM potassium phosphate monobasic, 15 mM ammonium sulfate, 1 mM magnesium sulfate, 1.7 µM ferrous sulfate adjusted to pH 7.0 with potassium hydroxide and supplemented with 0.3% glucose and 0.5% casamino acids) was used for all experiments unless stated otherwise [17]. LB media was 1% Bacto Tryptone, 0.5% yeast extract, and 0.5% sodium chloride. Antibiotics were used as needed at the following concentrations unless stated otherwise: 8 µg/ml for tobramycin, 200 µg/ml for carbenicillin, and 100 µg/ml for gentamicin. Tobramycin was stored at -20°C in single use aliquots. Strains and plasmids are listed in Tables S1 and S2, respectively. All *P. aeruginosa* mutants were in the MPAO1 strain background. All growth curves were done in a SynergyMx plate reader (Biotek); see Protocol S1 for details.

### Transposon Construction and Mutagenesis

Transposon mutagenesis of strain MPAO1 (SAH001) was carried out via a bi-parental conjugation with an *E. coli* S17**–**1 λ-pir donor strain carrying the *mariner* transposon construct on plasmid pBTK-*MAR2xT7*. Based on estimates from plating small aliquots immediately after the cells were scraped off the mating plates, the library contained ∼2×10^6^ independent transposon insertion mutants.

### Library Enrichments

Planktonic and biofilm experiments were all started with ∼1×10^8^ cells from the transposon library. In all planktonic experiments, tubes were shaken at 250 rpm; for biofilm enrichments, a sterile, plastic slide was provided as the biofilm formation substrate and cultures were not shaken. Approximately 4.7×10^6^ cells colonize every square millimeter of the slide (standard error  = 1.3×10^6^ of three experimental replicates). All the experiments were carried out at 37°C. For each round of enrichment, cultures were grown for 24 hours without tobramycin (initiation phase). Then a fraction of the old culture (for planktonic experiments) or the slide (for biofilm experiments) was transferred to fresh media either with or without tobramycin and grown for an additional 24 hours (selection phase). After 24 hours, a similar transfer was done to fresh media with no drug (recovery phase). This was followed by a second round of selection and recovery phases. Enriched populations were harvested by centrifugation and stored at -80°C. For biofilm samples, cells were removed from the slides by vigorous shaking and vortexing prior to centrifugation. For more details, see Protocol S1.

### Genetic Footprinting and Sample Preparation for Microarray Hybridization

DNA manipulations were similar to those described before [23] with some alterations. In brief, genomic DNA was isolated from frozen cell pellets using the QIAamp DNA Mini Kit (Qiagen), digested with a combination of BsaHI/ClaI/BstBI/AclI, NarI/HpyCH4IV, and HinP1I in three parallel reactions, and ligated to a Y-shaped linker [23]. Next, the ligation product was used as a template to amplify the DNA adjacent to both ends of the transposon. PCR product was transcribed *in vitro* using T7 RNA polymerase, reverse transcribed into biotin-labeled cDNA, fragmented to approximately 50**–**200 bp using DNase I, and hybridized to GeneChip *P. aeruginosa* Genome Arrays (Affymetrix). More detail is provided in the Protocol S1 document.

### Analysis of Microarray Footprinting Data

Probes that contained the recognition sites of restriction enzymes belonging to at least two of the following restriction enzyme sets (BsaHI/ClaI/BstBI/AclI, NarI/HpyCH4IV, or HinP1I) were excluded from the analysis. The signal for each gene was the average of the perfect match minus mismatch differences from the rest of the probes for the gene. Data from different arrays were sum-normalized prior to comparison (Dataset S1).

### Competition Assays

The identities of mutants from the UW collection [35] were verified by PCR using one primer from the transposon and one from the *P. aeruginosa* genome. Different mutants and the reference strain were labeled with a chromosomal copy of e-yfp or e-cfp, respectively, using broad host-range mini-Tn7 vectors [52]. To start a competition, roughly equal numbers of the CFP-labeled reference strain (SAH349) and the YFP-labeled mutant were mixed in a 2 ml tube. For planktonic competitions, the tube was shaken at 250 rpm; for biofilm competitions, the tube contained a piece of plastic slide and was not shaken. Similar to the library enrichment procedure, cells were taken through one cycle of initiation, selection, and recovery. At the end of the third day (i.e., the recovery phase), cells were harvested and grown to late exponential phase in order to obtain sufficient signal and to minimize the contribution of non-viable cells. Finally, the CFP (excitation: 433 nm, emission: 475 nm) and YFP (excitation: 510 nm, emission: 532 nm) signals were measured in the culture, using a SynergyMx plate reader to determine the abundance of each strain in the population. See the Protocol S1 document for more details.

### Disk Susceptibility Assays

Tobramycin impregnated disks (BD product #231569) were placed on LB plates that had been spread with 200 µl of overnight LB-grown cultures that had been diluted 100-fold. Zones of inhibition were measured after 24 h of incubation at 37°C.

### NAD Cycling Assay

Overnight, M63-grown cultures were diluted 1∶100 into fresh M63. When the cultures reached mid-log phase, two 1 ml samples, one each for NAD^+^ and NADH extractions, were harvested by spinning in a table-top centrifuge at maximum speed for 30 seconds. Supernatant was removed, and pellets were snap-frozen in an ethanol-dry ice bath. The Fluoro NAD™ kit (Cell Technology Incorporation) was used to determine NAD^+^ and NADH content, according to the manufacturer's instructions.

### Tobramycin-Outer Membrane Interaction Study

Cells were harvested from 1 ml mid-log phase cultures by centrifugation and re-suspended in 5 mM HEPES buffer, pH 7.2, supplemented with 5 µM carbonyl cyanide *m*-chlorophenylhydrazone. NPN (final concentration of 50 µM) and tobramycin (final concentration of 8 or 0 µg/ml) were added after 1 and 3 minutes, respectively, and incorporation of NPN into the membrane was measured in a SynergyMx plate reader using an excitation wavelength of 350 nm and an emission wavelength of 420 nm.

### Microarray Expression Meta-Analysis

*P. aeruginosa* expression datasets were downloaded from the GEO (Gene Expression OmniBus) database (See the Protocol S1 document for the full list). All the expression datasets were sum normalized, and missing values were estimated using a weighted K-nearest neighbor method (KNNimpute) [53]. Then, for each gene, *x*, in the genome and each gene, *y*, from Figure 3A and 3B, the Pearson's correlation coefficient between the expression profiles for×and *y* was calculated. Finally, the correlation coefficients were clustered using a K-means algorithm with a Euclidean distance metric [54].

### Expression Analysis

RNA was isolated from mid-exponential phase cultures of SAH084, SAH087, SAH108, and SAH502, converted to cDNA, fragmented, labeled with biotin, and hybridized to Affymetrix GeneChip *P. aeruginosa* Genome Arrays. Additional details are provided in Protocol S1. Complete data is provided in Dataset S2 and also deposited in the Gene Expression Omnibus (GEO) database with the accession number GSE26142.

### iPAGE Analysis

iPAGE was run locally using GO categories from Pseudocyc [55] and GOanna [56].

### Accession Numbers

Expression data from this work are archived in the GEO database with accession number GSE26142.

NCBI accession numbers for the genes and proteins mentioned in the text are provided below:

PA0614, 880722; PA0748, 879324; PA1329, 880896; *lasR*, 881789; *pscJ*, 881901; PA1732, 878043; *rbsB*, 878276; *mexE*, 880212; *nuoA*, 882344; *nuoK*, 882355; PA2653, 882362; PA2771, 882750; PA3048, 882879; PA3222, 882553; *rhlI*, 878967; PA3726, 880374; *purT*, 880455; PA3844, 879831; PA3966, 878878; PA4175, 880208; PA4516, 881122; PA5207, 879542; *wzm*, 883118.

## Supporting Information

Dataset S1 **Hybridization signals from transposon library enrichments and the original, unselected library.**
(XLS)

Dataset S2 **Mid-exponential phase expression data from the PA3726 mutant (SAH108) and three MPAO1 isolates.**
(XLS)

Dataset S3 **Clusters of genes whose modulation or disruption by transposons differentially altered fitness among different experimental conditions.**
(XLS)

Figure S1 **Viability of planktonic and biofilm cultures exposed to tobramycin.** Both planktonic and biofilm samples were started with 1∶100 dilutions of overnight SAH001 cultures in M63 media. In all planktonic experiments, tubes were shaken at 250 rpm; for biofilm experiments, a piece of sterile, plastic slide was provided as the biofilm formation substrate and cultures were not shaken. All the experiments were carried out at 37°C in 1 ml of media in a close 2 ml microfuge tube. Cultures were grown for 24 hr without tobramycin (initiation phase). Then, 10 µl of the culture (for planktonic experiments) or the slide (for biofilm experiments) was transferred to 1 ml of fresh media either with or without tobramycin and grown for an additional 24 hr (selection phase). After 24 hr, cells were harvested (for biofilm samples, cells were removed from the slides by vigorous shaking and vortexing) and cell counts were acquired by plating serial dilutions on LB plates. The reported number is the average of 5 experimental replicates for each sample, which is in a total volume of 1 ml. Error bars correspond to the standard error. CFU: colony-forming units.
(PDF)

Figure S2 **Competitive enrichment data for 45 genes selected for further analysis.** Shown is the genome-wide footprinting data for the 45 genes chosen for further analysis. See the ‘Competition Assays' section of Protocol S1 for selection criteria. Genes (rows) were arranged using hierarchical clustering, and the hybridization scores shown for each gene were mean-centered and normalized to a standard-deviation of one. Column labels indicate the experimental condition: Bio-ND and Bio-TOB refer to transposon insertion libraries grown as a biofilms and treated with no drug or tobramycin, respectively, and Pla-ND and Pla-TOB refer to libraries grown planktonically without or with tobramycin. Two biological replicates were performed in each condition, and numbers indicate the repetition number. Yellow (blue) indicates that transposon insertions were beneficial (deleterious) in the experimental condition.
(PDF)

Figure S3 **Fitness in Bio-TOB competitions of candidate mutants not chosen for further analysis.** Competitions started with equal amounts of mutant and reference cells. The y-axis indicates the relative amounts of cells following the experimental challenge (as explained in Methods). Error bars indicate the standard error of at least 8 experiments.
(PDF)

Figure S4 **Growth curves with 0** µ**g/ml tobramycin.** Shown are growth curves for all strains in Figures 3A and 3B in the absence of tobramycin.
(PDF)

Figure S5 **Growth curves with 4** µ**g/ml tobramycin.** Shown are growth curves for all strains in Figures 3A and 3B with 4 µg/ml tobramycin.
(PDF)

Figure S6 **Growth curves with 8** µ**g/ml tobramycin.** Shown are growth curves for all strains in Figures 3A and 3B with 8 µg/ml tobramycin.
(PDF)

Figure S7 **Kill curves in the planktonic and biofilm states.** Both planktonic and biofilm tests were conducted at 37°C in closed 2 ml microfuge tubes with 1 ml of media. Experiments were started with 1∶100 dilutions of overnight cultures grown in M63 media. (A) For the planktonic experiments, overnight cultures were added to fresh media with 8 µg/ml of tobramycin (or 0 µg/ml tobramycin for the no drug control). Tubes were shaken at 250 rpm and viability was measured at the indicated times by plating serial dilutions of the cultures. (B) For biofilm experiments, a sterile, plastic slide was provided as the biofilm formation substrate and cultures were not shaken. Cultures were grown for 24 hr without tobramycin to allow biofilms to form. Then, the slide was transferred to 1 ml of fresh media with 8 µg/ml tobramycin (or 0 µg/ml tobramycin for the no drug control) and grown for the indicated time. To harvest the biofilm samples, the slides were moved into PBS and the cells were removed from the slides by vigorous shaking and vortexing. Cell counts were acquired by plating serial dilutions on LB plates. Numbers are the average of at least 3 and 4 experimental replicates for the planktonic and biofilm settings, respectively. Error bars show the standard error. CFU: colony-forming units, ND: no drug. The following strains were used: SAH020 (PA0748), SAH027 (PA2646, *nuoK*), SAH032 (PA2771), MPAO1 (SAH084), SAH110 (PA4516), SAH114 (PA2653), and SAH129 (PA3966).
(PDF)

Figure S8 **Complete data for NAD cycling assay.** The NADH/NAD^+^ ratio was measured in the following mutants that had the most pronounced growth advantage in the planktonic phase: SAH018 (PA1329), SAH041 (PA3476, *rhlI*), SA087 (MPAO1), SAH116 (PA3048), SAH124 (PA3844), SAH128 (PA3222), SAH129 (PA3966), SAH130 (PA5207), and SAH027 (PA2646, *nuoK*).
(PDF)

Figure S9 **Complete data for tobramycin-outer membrane interaction assay.** As explained in the Methods section, the interaction of tobramycin with the outer membrane was measured using an NPN assay in the following strains: SAH018 (PA1329), SAH020 (PA0748), SAH032 (PA2771), SAH087 (MPAO1), SAH110 (PA4516), SAH112 (PA1732), SAH114 (PA2653), SAH116 (PA3048), SAH121 (PA2493, *mexE*), SAH124 (PA3844), SAH127 (PA1723, *pscJ*), SAH128 (PA3222), SAH129 (PA3966), SAH130 (PA5207), SAH318 (PA1946, *rbsB*), SAH320 (PA3751, *purT*), and SAH328 (PA4175). NPN (final concentration of 50 µM) and tobramycin (final concentration of 8 µg/ml) were added after 60 and 180 seconds, respectively. Error bars show the standard deviation of the data from all the mutants shown.
(PDF)

Figure S10 **Complete data for** ***lasR*** **reporter activity.** Quorum sensing reporter plasmid pGJB6 (*rsaL:gfp*, *lasR* reporter) was used to monitor the activity of the *las* quorum sensing system in all mutants except those whose annotations suggested a low chance of quorum-sensing involvement.
(PDF)

Figure S11 **Complete data for** ***rhlR*** **reporter activity.** Quorum sensing reporter plasmid pYL121 (*rhlAB:gfp*, *rhlR* reporter) was used to monitor the activity of the *rhl* quorum sensing system in all mutants except those whose annotations suggested a low chance of quorum-sensing involvement.
(PDF)

Figure S12 **Expression data places** ***P. aeruginosa*** **genes into three global classes.** Each row corresponds to a gene in the *P. aeruginosa* genome for which expression data was available; each column corresponds to a gene whose disruption was confirmed to affect fitness in the Bio-TOB setting (Figure 3A, 3B). The colors represent the Pearson's correlation coefficient of the expression profiles for the pair of genes.
(PDF)

Figure S13 **iPAGE meta-analysis of expression data.** Using iPAGE, we searched for functional enrichments or depletions in each of the three classes from Figure S12.
(PDF)

Figure S14 **Genes whose disruption alters Bio-TOB fitness are not all co-expressed.** As in Figure S12, colors represent the Pearson's correlation coefficient of the expression profiles for the pair of genes. Genes were arranged by hierarchical clustering. Shown are the genes whose disruption by transposon, substantially altered fitness in the Bio-TOB setting (Figure 3A, 3B).
(PDF)

Figure S15 **Functional relationships between PA2771 expression and genome-wide expression.** Pearson's correlation coefficients comparing the expression of all genes to the expression of PA2771 were subjected to iPAGE analysis to detect over- and under-represented functional categories in each range of correlation. Expression data came from the same 255 published expression arrays used for Figures S12, S13, and S14.
(PDF)

Figure S16 **Ciprofloxacin induction of PA0614 promoter.** In order to control for the functionality of the PA0614'-*gfp* construct, MPAO1 cells carrying pUCP20-PA0614'-*gfp* plasmid were grown in the presence of different ciprofloxacin concentrations and (A) the promoter activity (*gfp* fluorescence) and (B) the culture density (absorbance) were measured.
(PDF)

Figure S17 **PA3057 promoter activity.** PA3057 promoter activity was measured using a *gfp* fusion reporter in both biofilm and planktonic settings in the presence or absence of 8 µg/ml tobramycin. No significant difference was observed in the promoter activity in any of these different settings. Promoter activities are normalized by colony forming units (CFU).
(PDF)

Protocol S1 **A more detailed description of the protocols and methods.**
(DOC)

Table S1 **Strain table.** List of all strains used in this study.
(XLS)

Table S2 **Plasmid table.** List of all plasmids used in this study.
(XLS)

Table S3 **Summary of genes with altered fitness in the Bio-TOB assay that were subjected to further characterization.** Shown are the PA#s, annotations, and predicted pathway of action (if available) for genes whose disruptions alters fitness in the Bio-TOB assays of Figure 3A and 3B.
(XLS)
