# Supplementary figures and images for: Fitness Landscape of Antibiotic Tolerance in Pseudomonas aeruginosa Biofilms
Source: PLoS Pathog. 2011 Oct 20;7(10):e1002298. doi: 10.1371/journal.ppat.1002298 (PMC3197603; doi:10.1371/journal.ppat.1002298)

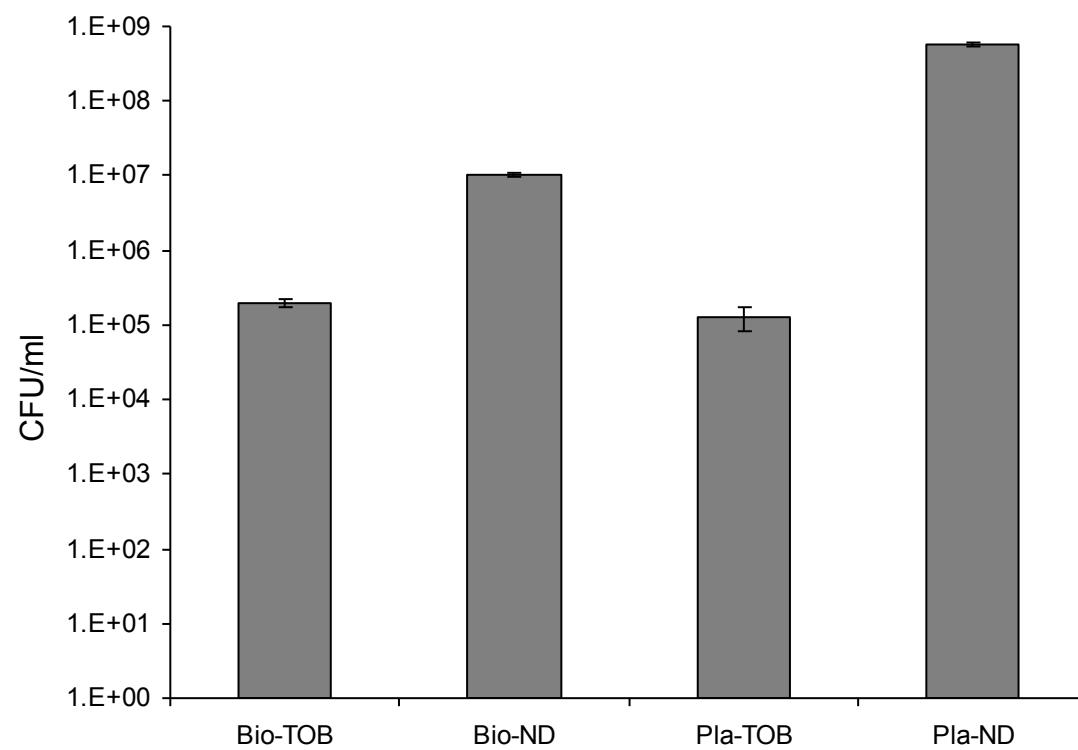

Supplement: Figure S1 — Viability of planktonic and biofilm cultures exposed to tobramycin. Both planktonic and biofilm samples were started with 1∶100 dilutions of overnight SAH001 cultures in M63 media. In all planktonic experiments, tubes were shaken at 250 rpm; for biofilm experiments, a piece of sterile, plastic slide was provided as the biofilm formation substrate and cultures were not shaken. All the experiments were carried out at 37°C in 1 ml of media in a close 2 ml microfuge tube. Cultures were grown for 24 hr without tobramycin (initiation phase). Then, 10 µl of the culture (for planktonic experiments) or the slide (for biofilm experiments) was transferred to 1 ml of fresh media either with or without tobramycin and grown for an additional 24 hr (selection phase). After 24 hr, cells were harvested (for biofilm samples, cells were removed from the slides by vigorous shaking and vortexing) and cell counts were acquired by plating serial dilutions on LB plates. The reported number is the average of 5 experimental replicates for each sample, which is in a total volume of 1 ml. Error bars correspond to the standard error. CFU: colony-forming units. (PDF) [file ppat.1002298.s004.pdf]

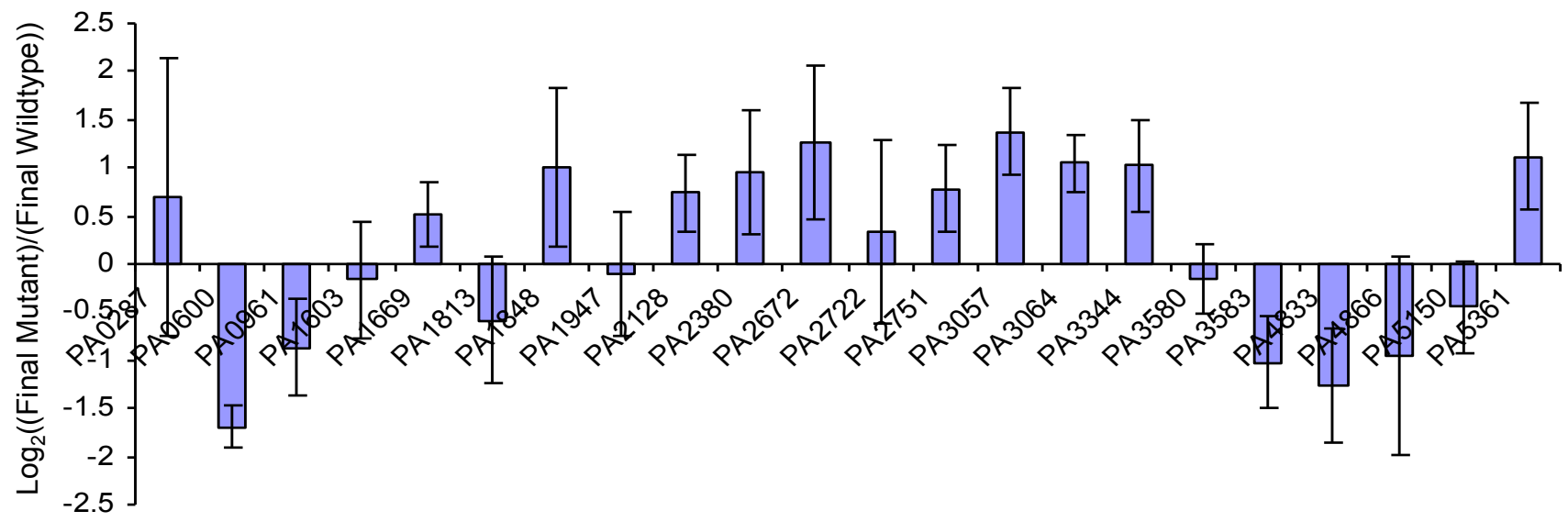

Supplement: Figure S3 — Fitness in Bio-TOB competitions of candidate mutants not chosen for further analysis. Competitions started with equal amounts of mutant and reference cells. The y-axis indicates the relative amounts of cells following the experimental challenge (as explained in Methods). Error bars indicate the standard error of at least 8 experiments. (PDF) [file ppat.1002298.s006.pdf]

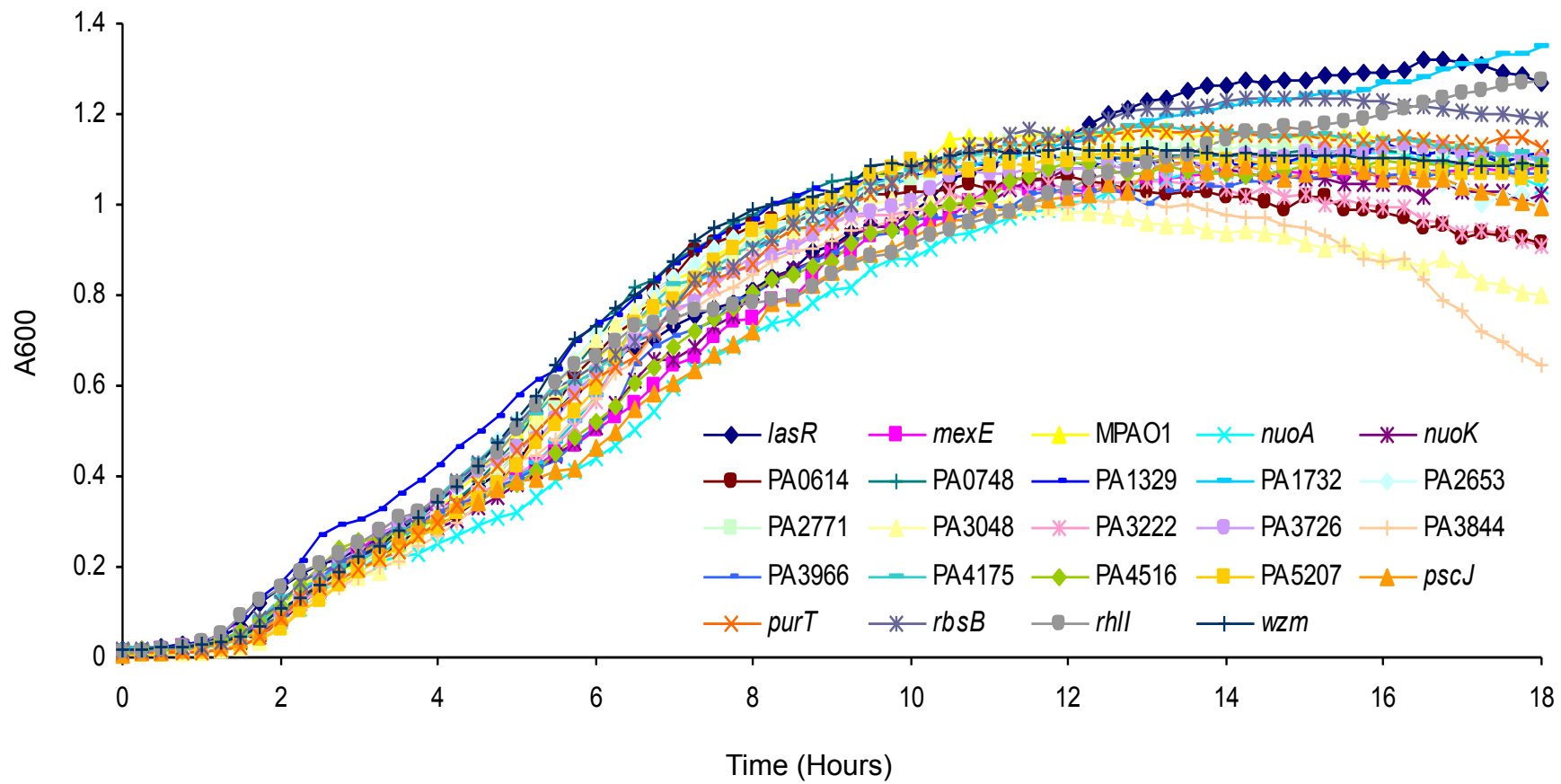

Supplement: Figure S4 — Growth curves with 0 µg/ml tobramycin. Shown are growth curves for all strains in Figures 3A and 3B in the absence of tobramycin. (PDF) [file ppat.1002298.s007.pdf]

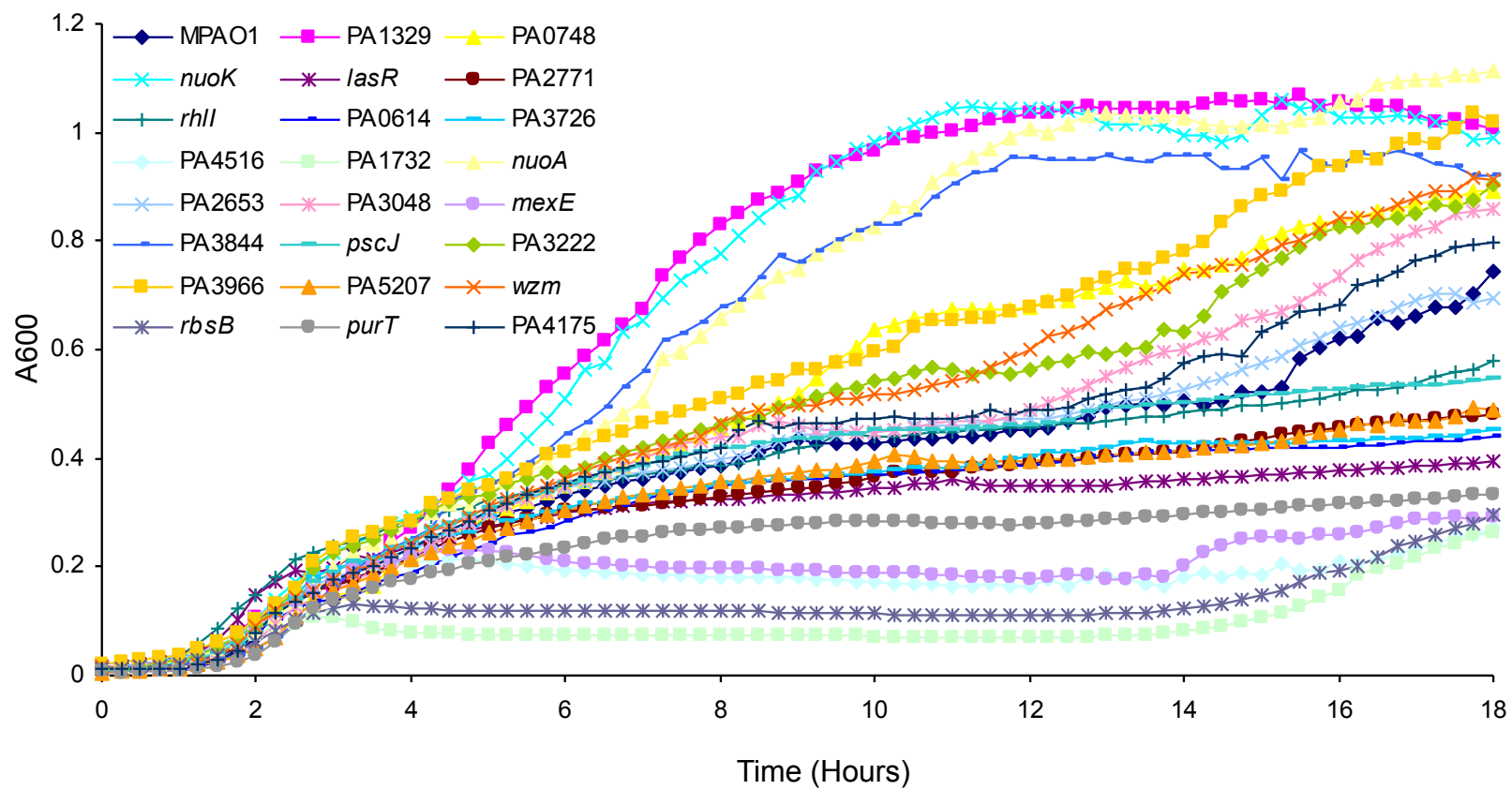

Supplement: Figure S5 — Growth curves with 4 µg/ml tobramycin. Shown are growth curves for all strains in Figures 3A and 3B with 4 µg/ml tobramycin. (PDF) [file ppat.1002298.s008.pdf]

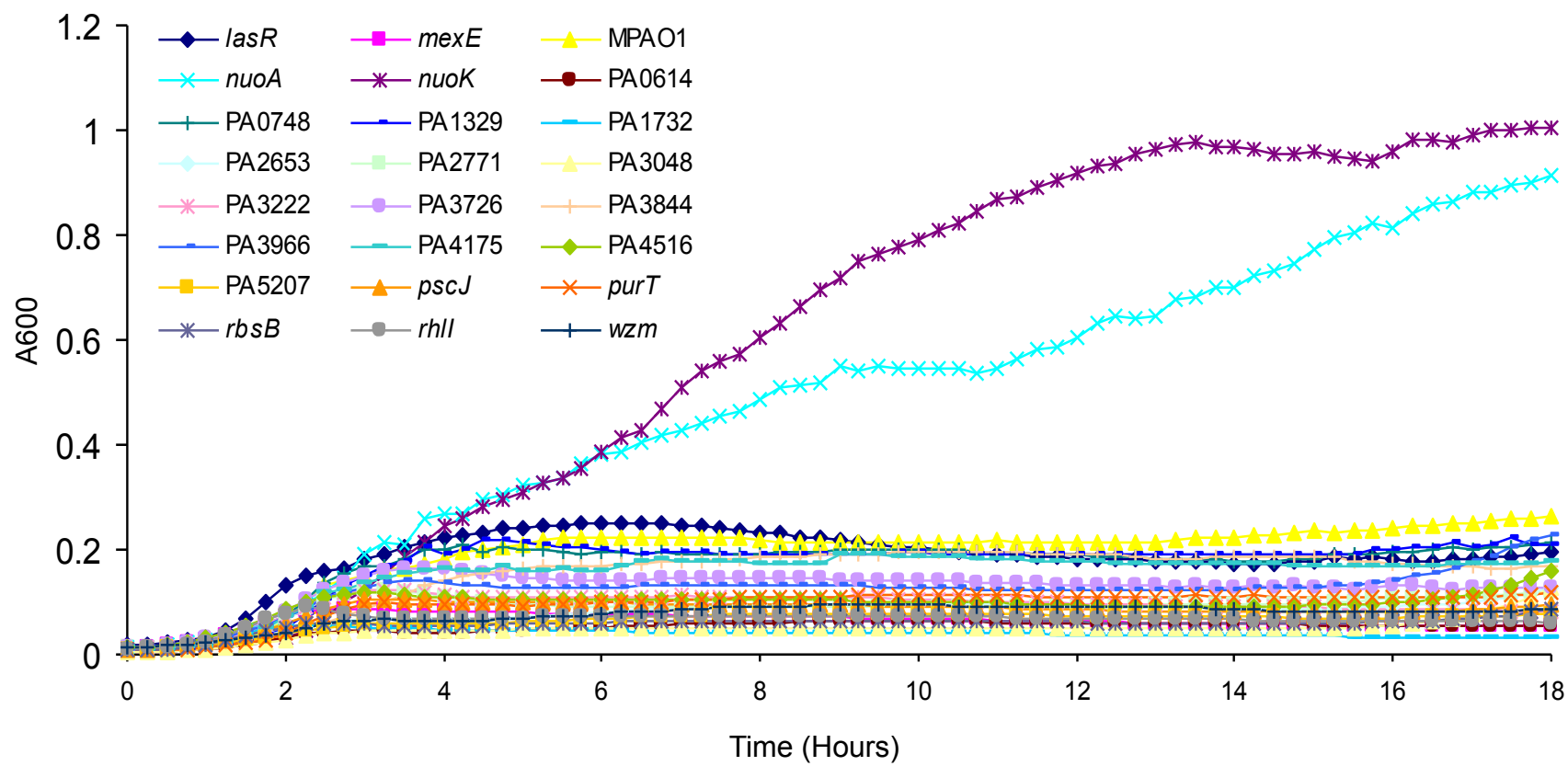

Supplement: Figure S6 — Growth curves with 8 µg/ml tobramycin. Shown are growth curves for all strains in Figures 3A and 3B with 8 µg/ml tobramycin. (PDF) [file ppat.1002298.s009.pdf]

**A**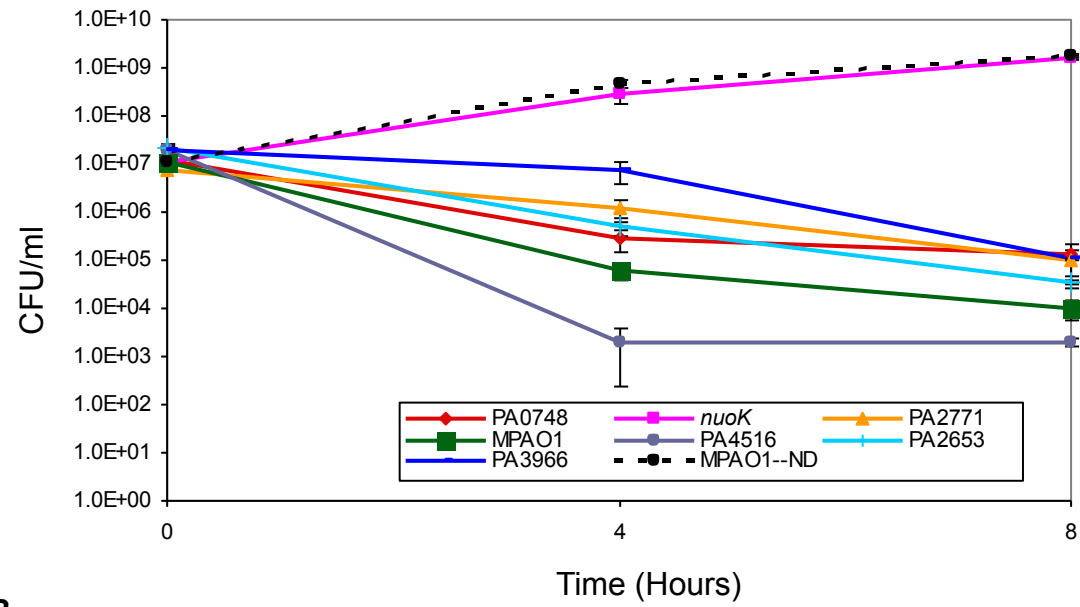**B**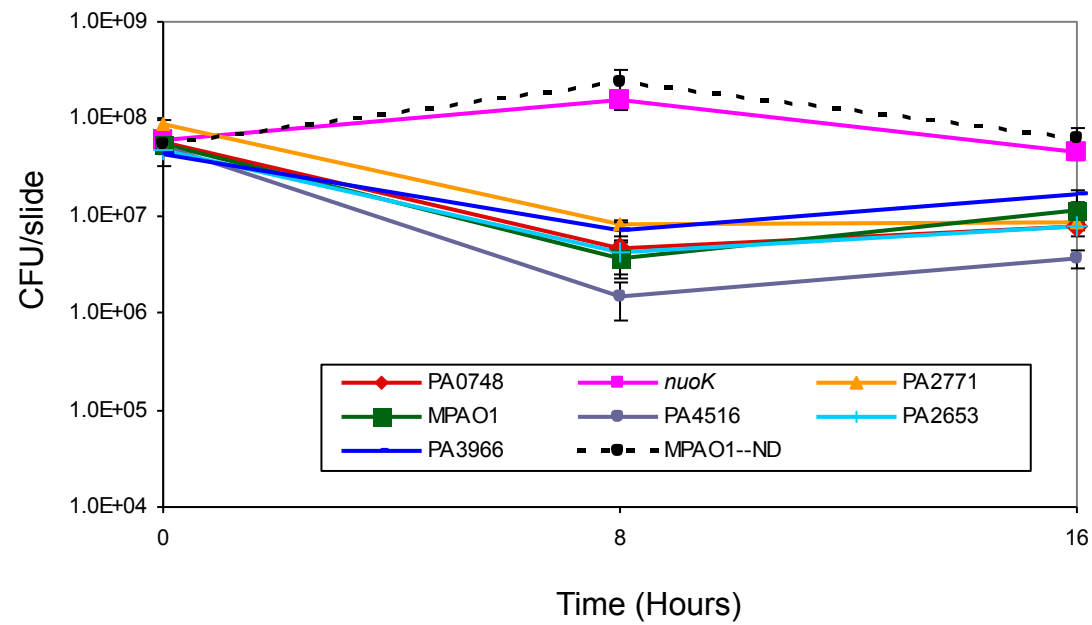

Supplement: Figure S7 — Kill curves in the planktonic and biofilm states. Both planktonic and biofilm tests were conducted at 37°C in closed 2 ml microfuge tubes with 1 ml of media. Experiments were started with 1∶100 dilutions of overnight cultures grown in M63 media. (A) For the planktonic experiments, overnight cultures were added to fresh media with 8 µg/ml of tobramycin (or 0 µg/ml tobramycin for the no drug control). Tubes were shaken at 250 rpm and viability was measured at the indicated times by plating serial dilutions of the cultures. (B) For biofilm experiments, a sterile, plastic slide was provided as the biofilm formation substrate and cultures were not shaken. Cultures were grown for 24 hr without tobramycin to allow biofilms to form. Then, the slide was transferred to 1 ml of fresh media with 8 µg/ml tobramycin (or 0 µg/ml tobramycin for the no drug control) and grown for the indicated time. To harvest the biofilm samples, the slides were moved into PBS and the cells were removed from the slides by vigorous shaking and vortexing. Cell counts were acquired by plating serial dilutions on LB plates. Numbers are the average of at least 3 and 4 experimental replicates for the planktonic and biofilm settings, respectively. Error bars show the standard error. CFU: colony-forming units, ND: no drug. The following strains were used: SAH020 (PA0748), SAH027 (PA2646, nuoK), SAH032 (PA2771), MPAO1 (SAH084), SAH110 (PA4516), SAH114 (PA2653), and SAH129 (PA3966). (PDF) [file ppat.1002298.s010.pdf]

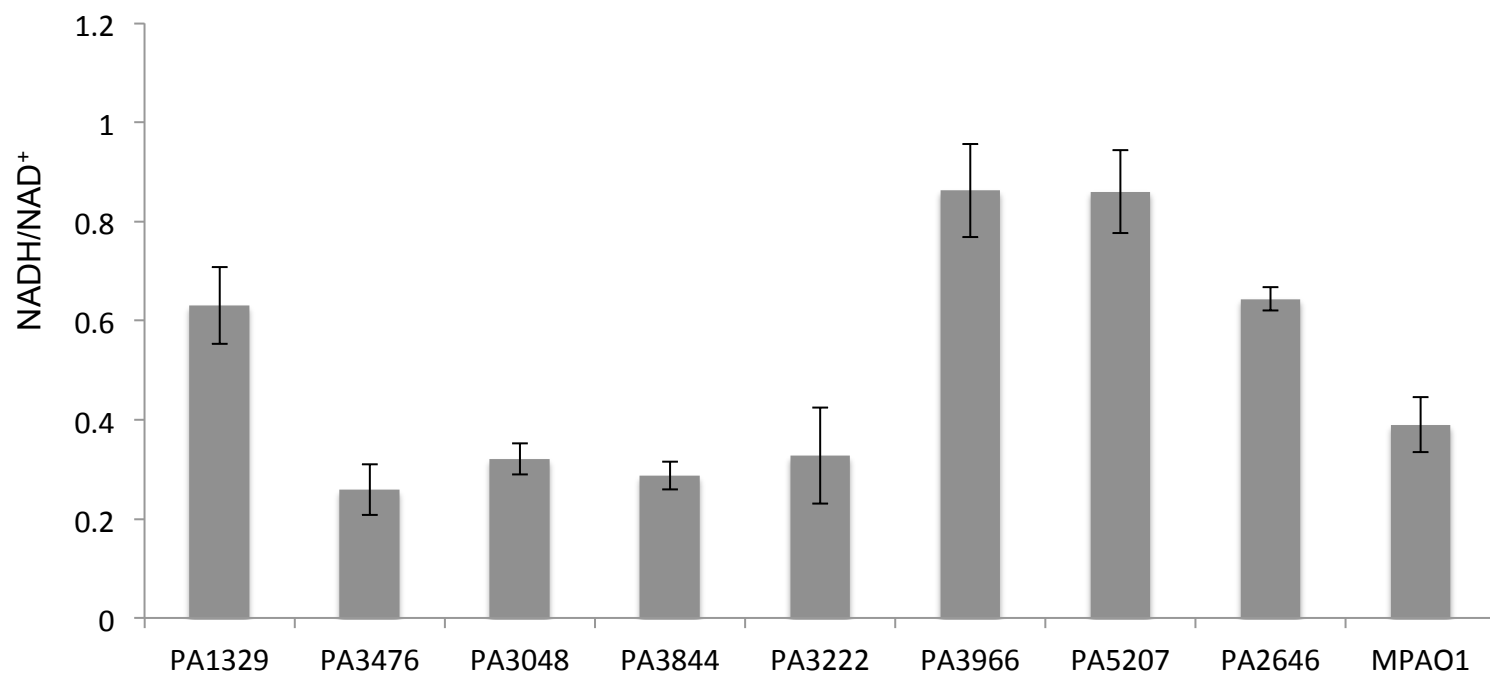

Supplement: Figure S8 — Complete data for NAD cycling assay. The NADH/NAD+ ratio was measured in the following mutants that had the most pronounced growth advantage in the planktonic phase: SAH018 (PA1329), SAH041 (PA3476, rhlI), SA087 (MPAO1), SAH116 (PA3048), SAH124 (PA3844), SAH128 (PA3222), SAH129 (PA3966), SAH130 (PA5207), and SAH027 (PA2646, nuoK). (PDF) [file ppat.1002298.s011.pdf]

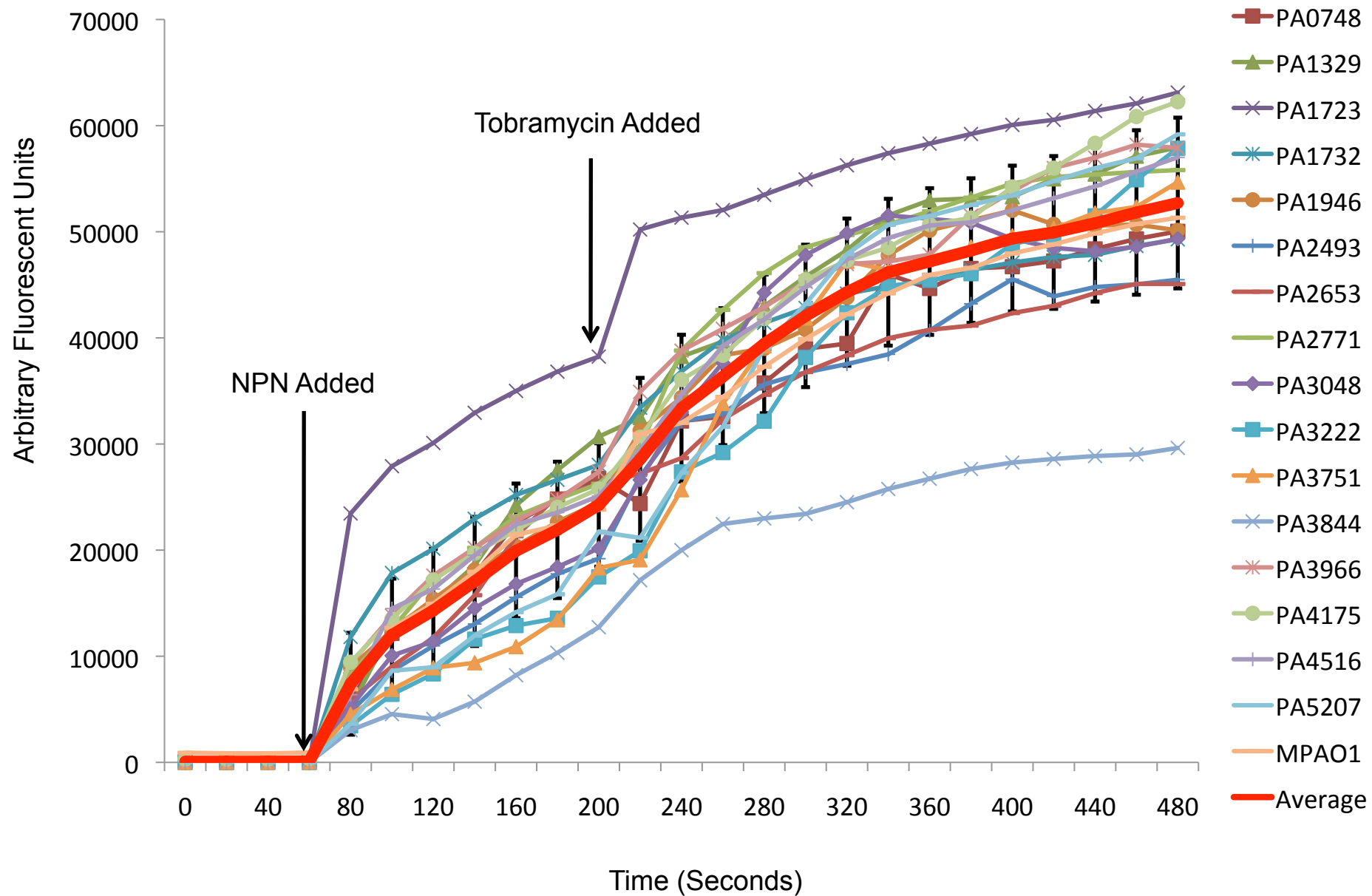

Supplement: Figure S9 — Complete data for tobramycin-outer membrane interaction assay. As explained in the Methods section, the interaction of tobramycin with the outer membrane was measured using an NPN assay in the following strains: SAH018 (PA1329), SAH020 (PA0748), SAH032 (PA2771), SAH087 (MPAO1), SAH110 (PA4516), SAH112 (PA1732), SAH114 (PA2653), SAH116 (PA3048), SAH121 (PA2493, mexE), SAH124 (PA3844), SAH127 (PA1723, pscJ), SAH128 (PA3222), SAH129 (PA3966), SAH130 (PA5207), SAH318 (PA1946, rbsB), SAH320 (PA3751, purT), and SAH328 (PA4175). NPN (final concentration of 50 µM) and tobramycin (final concentration of 8 µg/ml) were added after 60 and 180 seconds, respectively. Error bars show the standard deviation of the data from all the mutants shown. (PDF) [file ppat.1002298.s012.pdf]

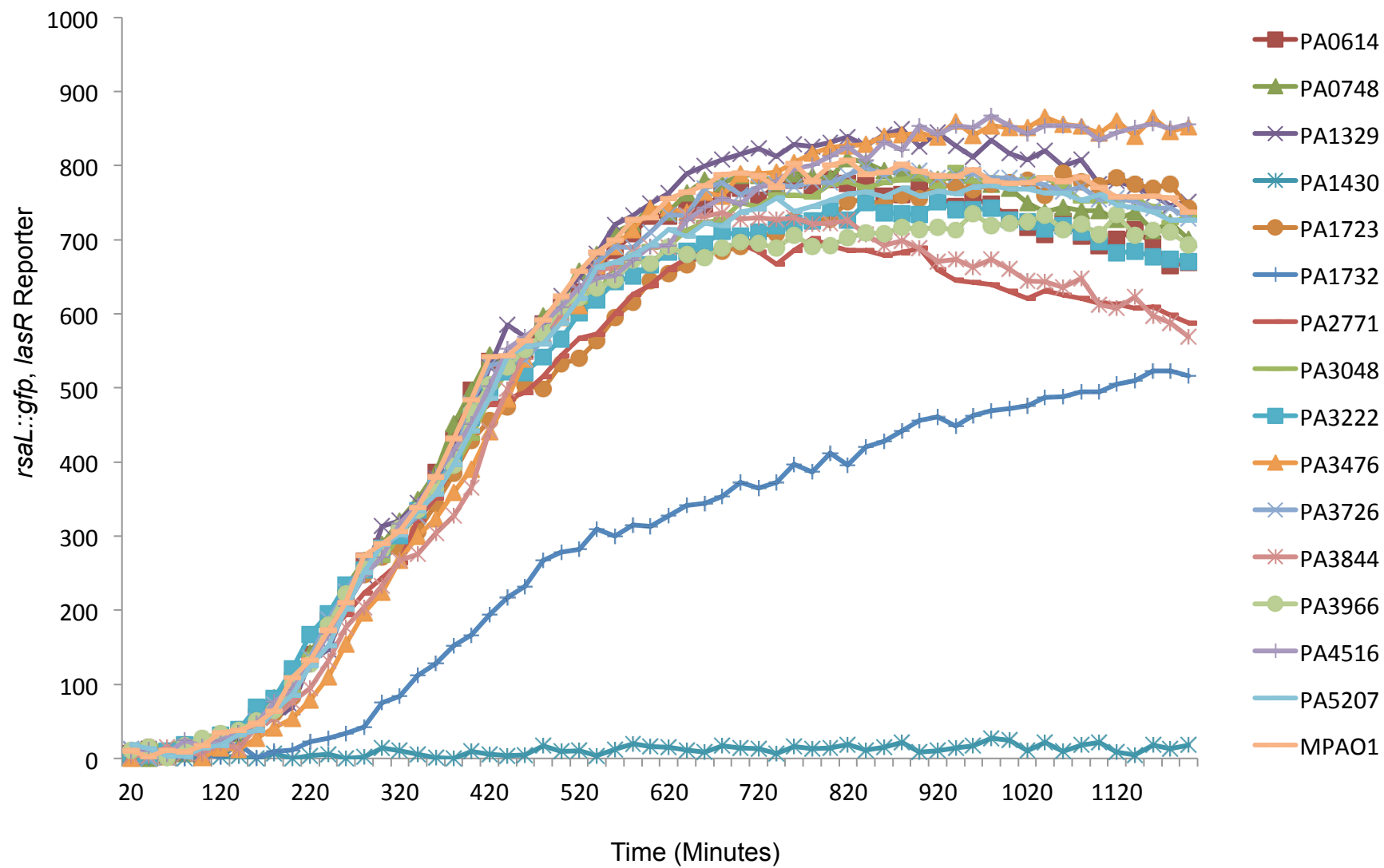

Supplement: Figure S10 — Complete data for lasR reporter activity. Quorum sensing reporter plasmid pGJB6 (rsaL:gfp, lasR reporter) was used to monitor the activity of the las quorum sensing system in all mutants except those whose annotations suggested a low chance of quorum-sensing involvement. (PDF) [file ppat.1002298.s013.pdf]

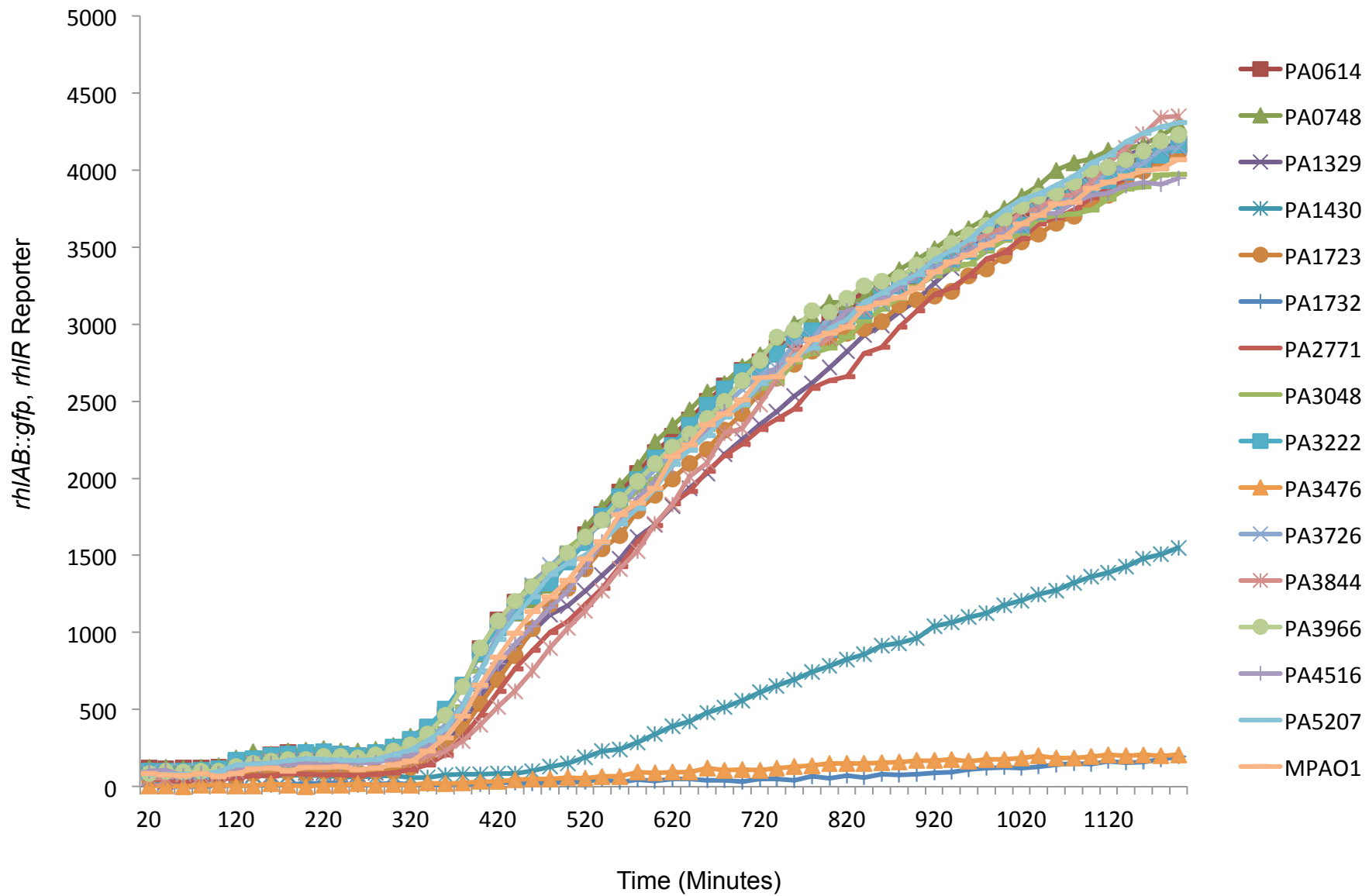

Supplement: Figure S11 — Complete data for rhlR reporter activity. Quorum sensing reporter plasmid pYL121 (rhlAB:gfp, rhlR reporter) was used to monitor the activity of the rhl quorum sensing system in all mutants except those whose annotations suggested a low chance of quorum-sensing involvement. (PDF) [file ppat.1002298.s014.pdf]

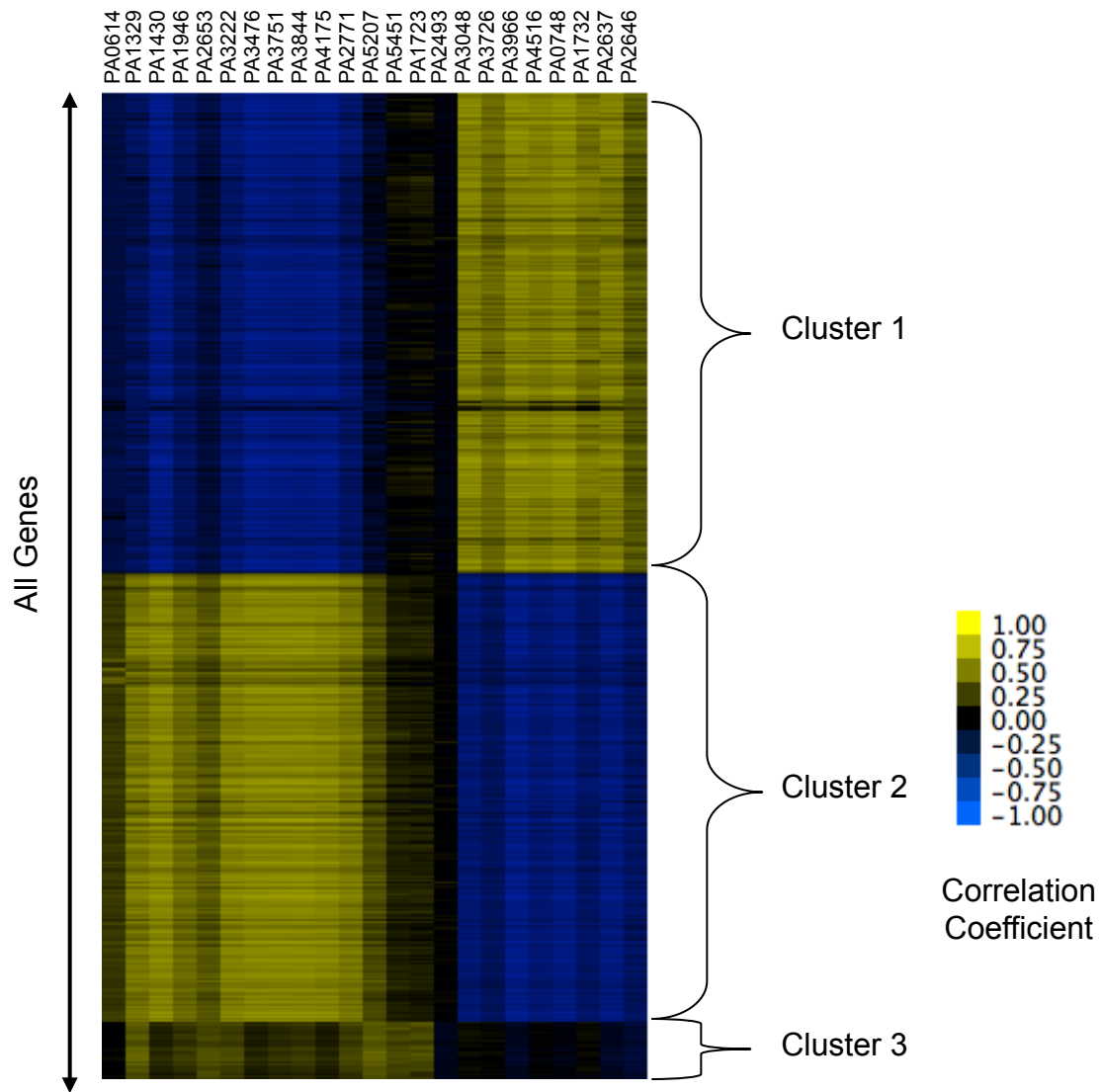

Supplement: Figure S12 — Expression data places P. aeruginosa genes into three global classes. Each row corresponds to a gene in the P. aeruginosa genome for which expression data was available; each column corresponds to a gene whose disruption was confirmed to affect fitness in the Bio-TOB setting (Figure 3A, 3B). The colors represent the Pearson's correlation coefficient of the expression profiles for the pair of genes. (PDF) [file ppat.1002298.s015.pdf]

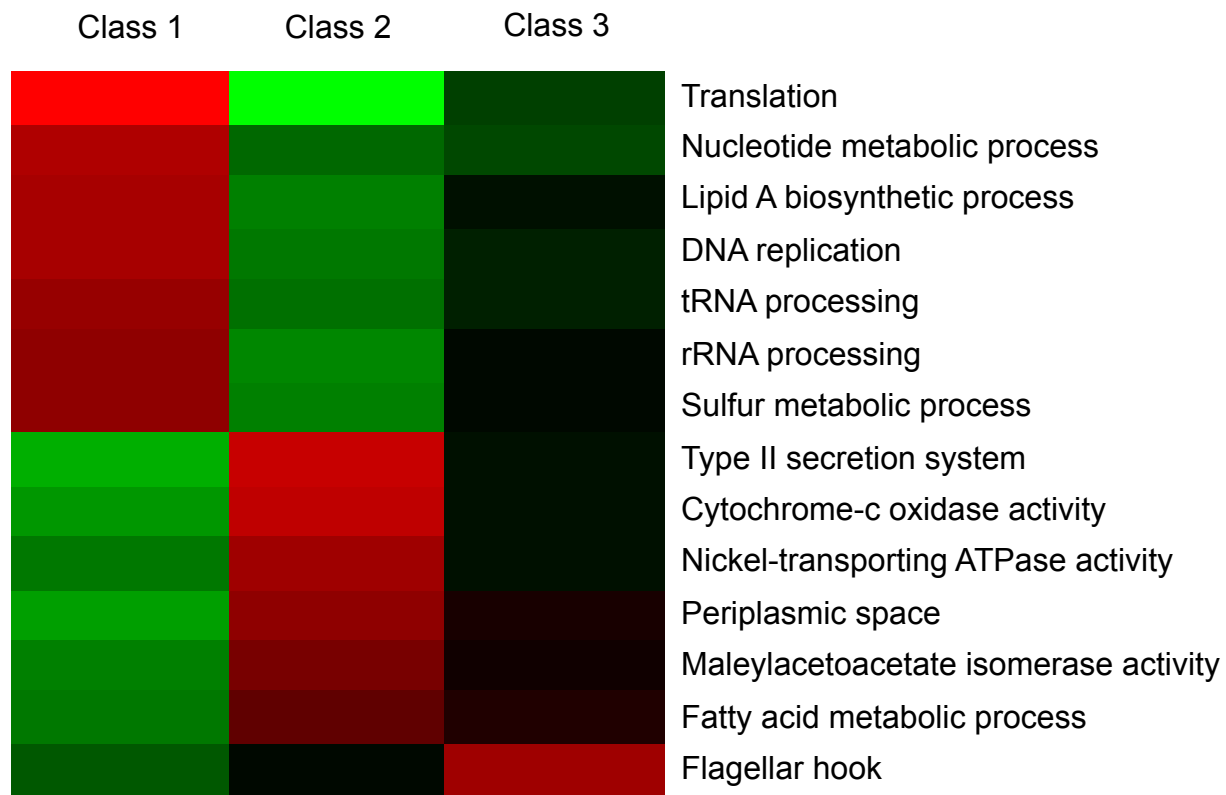

Under-representation      Over-representation  
 $-\text{Log}_{10}(\text{p-value}) = 6$        $-\text{Log}_{10}(\text{p-value}) = 6$

Supplement: Figure S13 — iPAGE meta-analysis of expression data. Using iPAGE, we searched for functional enrichments or depletions in each of the three classes from Figure S12. (PDF) [file ppat.1002298.s016.pdf]

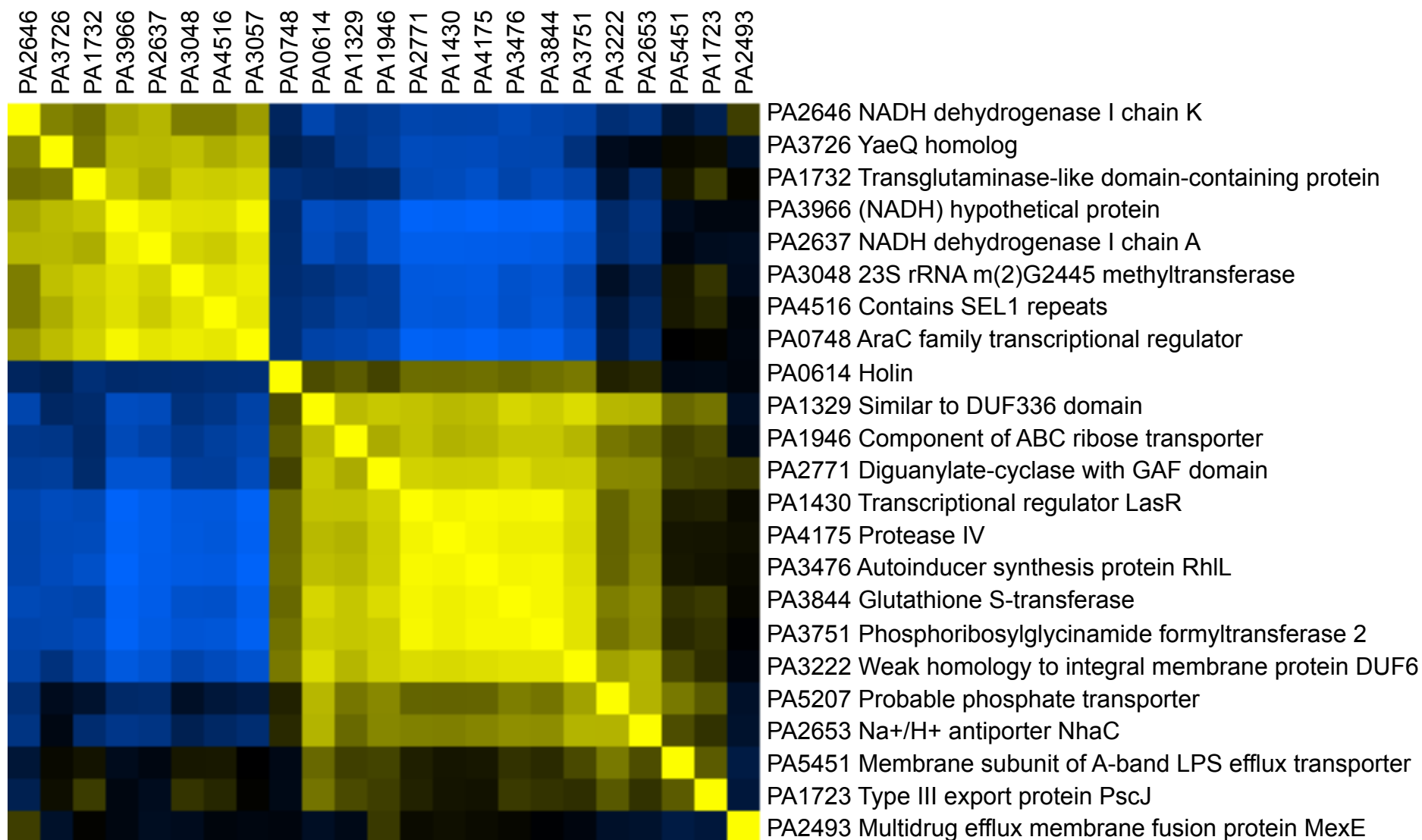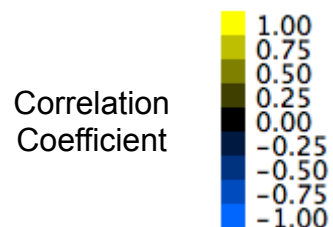

Supplement: Figure S14 — Genes whose disruption alters Bio-TOB fitness are not all co-expressed. As in Figure S12, colors represent the Pearson's correlation coefficient of the expression profiles for the pair of genes. Genes were arranged by hierarchical clustering. Shown are the genes whose disruption by transposon, substantially altered fitness in the Bio-TOB setting (Figure 3A, 3B). (PDF) [file ppat.1002298.s017.pdf]

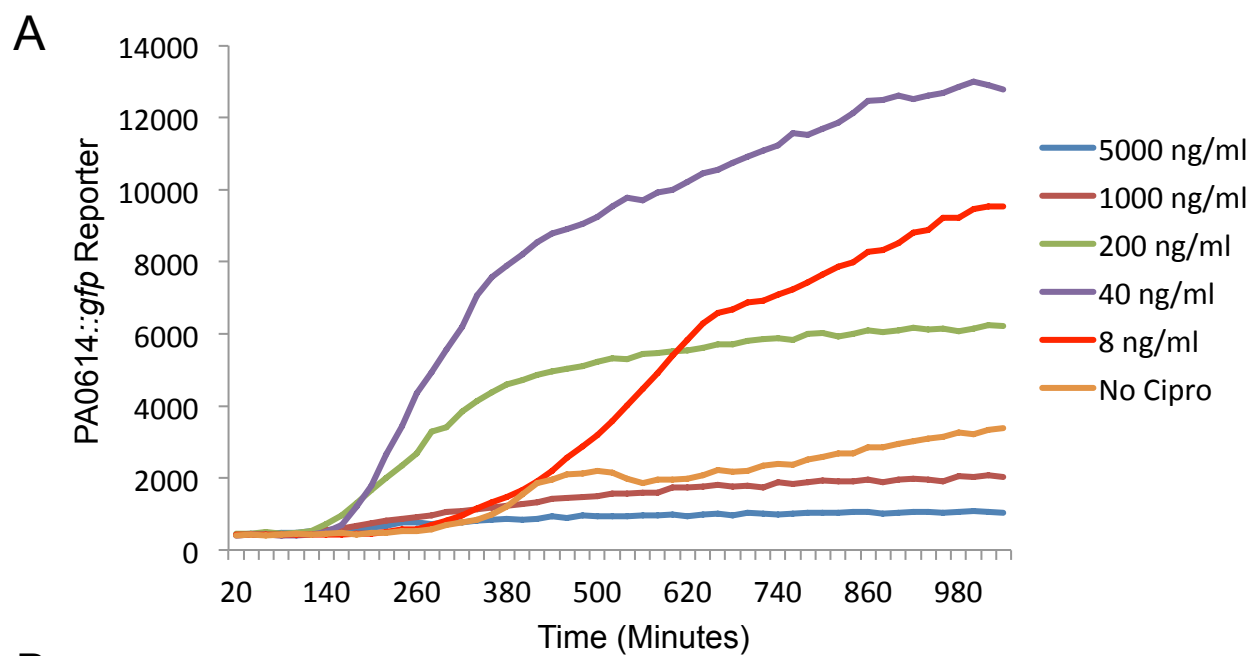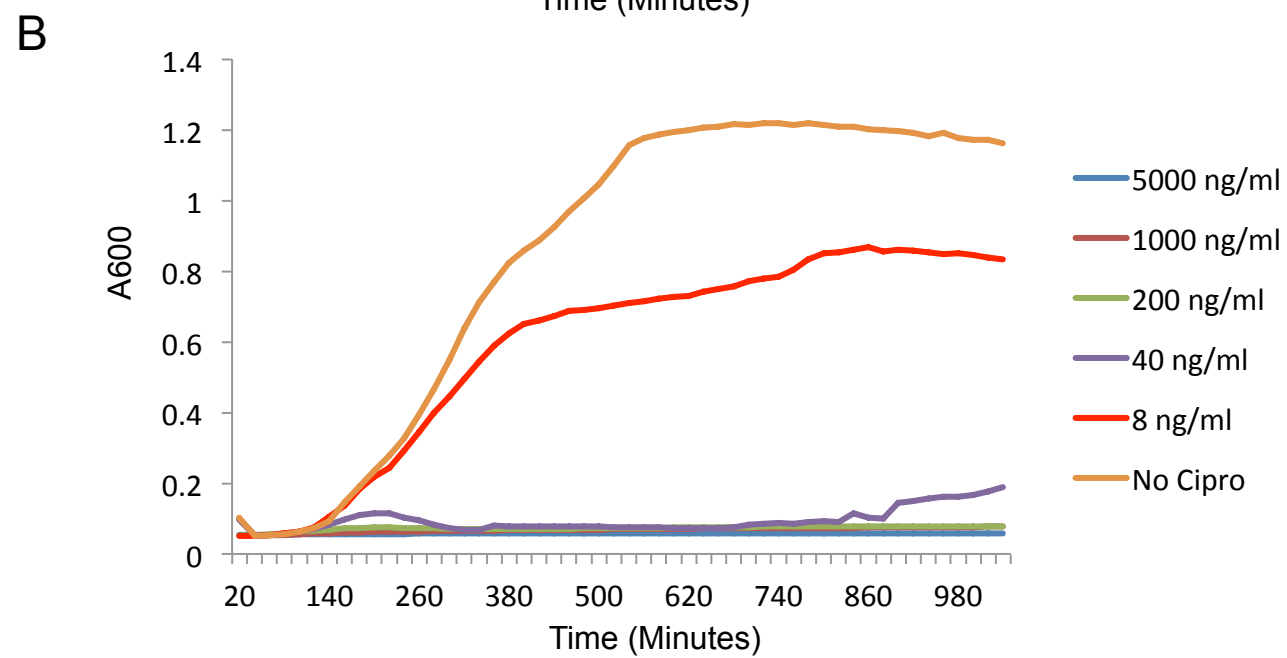

Supplement: Figure S16 — Ciprofloxacin induction of PA0614 promoter. In order to control for the functionality of the PA0614'-gfp construct, MPAO1 cells carrying pUCP20-PA0614'-gfp plasmid were grown in the presence of different ciprofloxacin concentrations and (A) the promoter activity (gfp fluorescence) and (B) the culture density (absorbance) were measured. (PDF) [file ppat.1002298.s019.pdf]

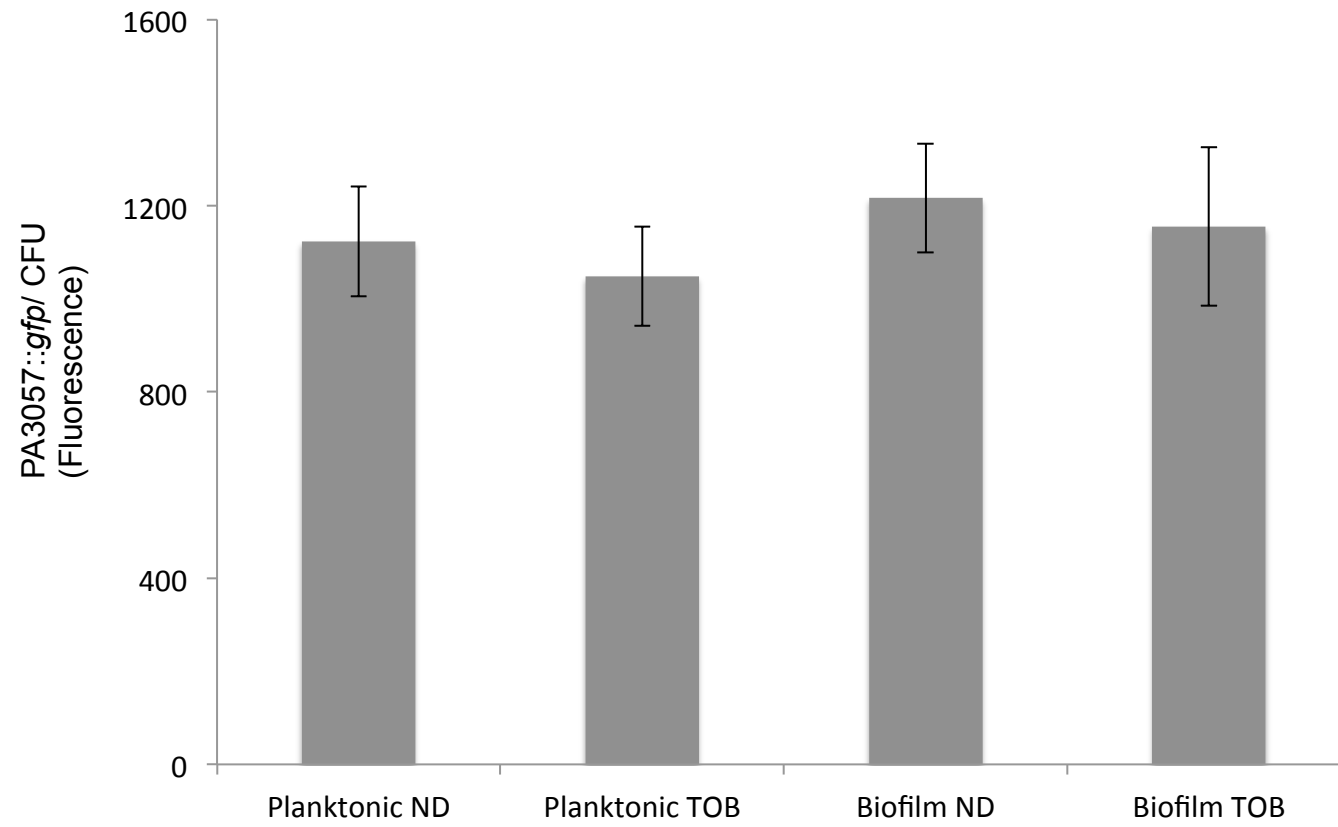

Supplement: Figure S17 — PA3057 promoter activity. PA3057 promoter activity was measured using a gfp fusion reporter in both biofilm and planktonic settings in the presence or absence of 8 µg/ml tobramycin. No significant difference was observed in the promoter activity in any of these different settings. Promoter activities are normalized by colony forming units (CFU). (PDF) [file ppat.1002298.s020.pdf]
