# Supplementary material for: Fitness Landscape of Antibiotic Tolerance in Pseudomonas aeruginosa Biofilms
Source: PLoS Pathog. 2011 Oct 20;7(10):e1002298. doi: 10.1371/journal.ppat.1002298 (PMC3197603; doi:10.1371/journal.ppat.1002298)
Supplement: Figure S15 — Functional relationships between PA2771 expression and genome-wide expression. Pearson's correlation coefficients comparing the expression of all genes to the expression of PA2771 were subjected to iPAGE analysis to detect over- and under-represented functional categories in each range of correlation. Expression data came from the same 255 published expression arrays used for Figures S12, S13, and S14. (PDF) [file ppat.1002298.s018.pdf]

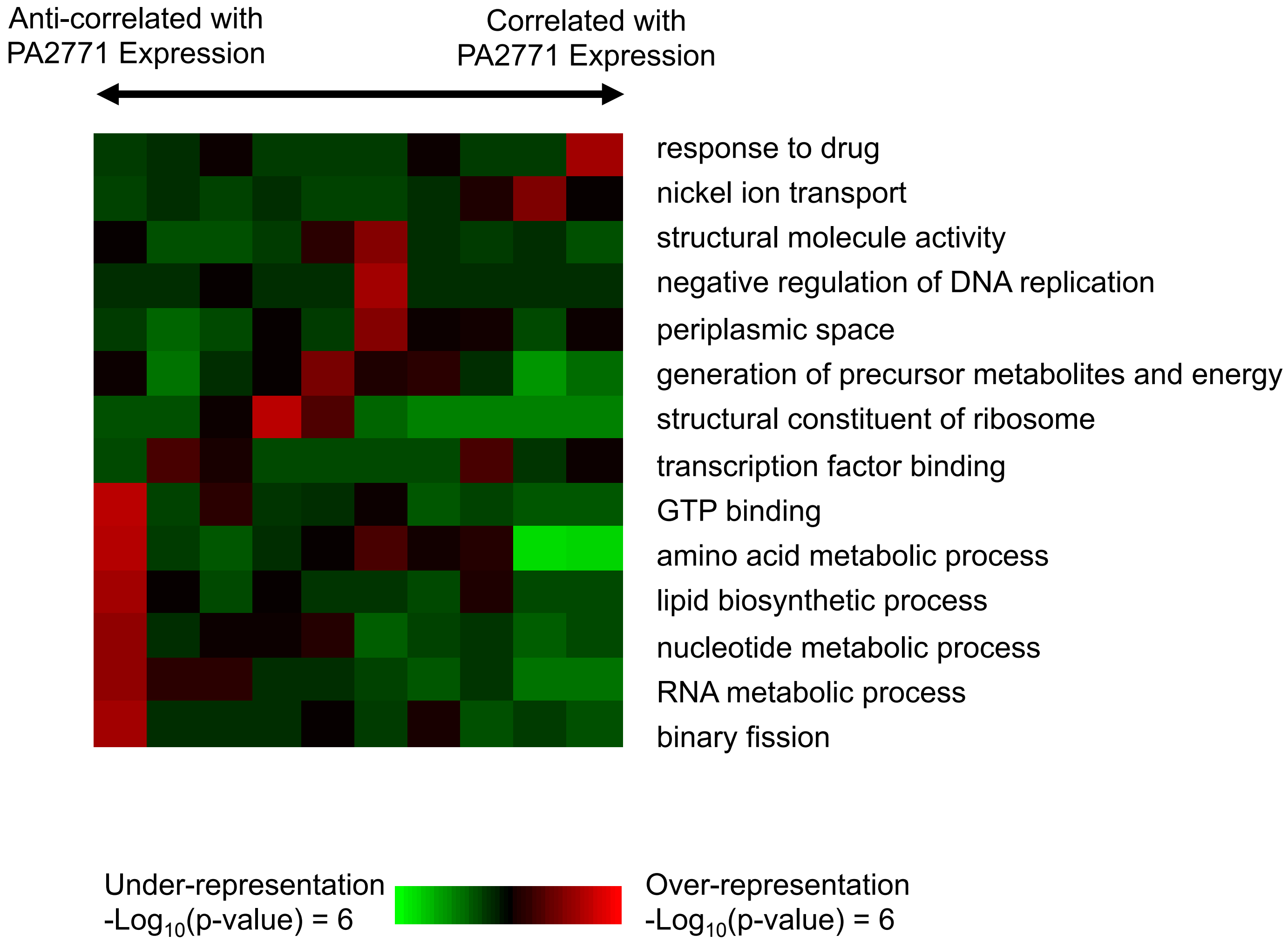
Anti-correlated with PA2771 Expression
Correlated with PA2771 Expression
response to drug
nickel ion transport
structural molecule activity
negative regulation of DNA replication
periplasmic space
generation of precursor metabolites and energy
structural constituent of ribosome
transcription factor binding
GTP binding
amino acid metabolic process
lipid biosynthetic process
nucleotide metabolic process
RNA metabolic process
binary fission
Under-representation -Log10(p-value) = 6
Over-representation -Log10(p-value) = 6
