# Supplementary material for: Fitness Landscape of Antibiotic Tolerance in Pseudomonas aeruginosa Biofilms
Source: PLoS Pathog. 2011 Oct 20;7(10):e1002298. doi: 10.1371/journal.ppat.1002298 (PMC3197603; doi:10.1371/journal.ppat.1002298)
Supplement: Protocol S1 — A more detailed description of the protocols and methods. (DOC) [file ppat.1002298.s021.doc]

**Protocol S1:**

**Transposon Construction and Mutagenesis**

The *mariner* transposon construct, which includes two outward-reading T7 promoters and an *aacC1* gene that confers gentamicin resistance, was cut from plasmid pMAR*2xT7* using SacI and SnaBI and placed into a pBTK30 backbone that had been digested by the same two restriction enzymes. The pBTK30 backbone has higher conjugation efficiency and hence is more suitable for high-yield transposon mutagenesis. The transposon was introduced to MPAO1 (SAH001) via conjugation using *E. coli* S17-1 λ-pir as the donor strain. To increase conjugation efficiency, MPAO1 recipient cells were grown at 42˚C in static LB (1% BactoTryptone, 0.5% yeast extract, 0.5% NaCl) cultures prior to conjugation . Then, donor and recipient cells were harvested by centrifugation, washed twice in PBS, and re-suspended in 100 mM MgSO_4_. Multiple mating spots from a 1:10 (original culture volumes) MPAO1: *E. coli* mixture were placed on LB plates and incubated at 37˚C for 2 hr. Next, cells were scraped off the plates and transferred to LB with 25 μg/ml of irgasan. After 20 min of recovery, gentamicin was added to a concentration of 50 μg/ml. Then, after another 20 min, additional gentamicin was added to bring the total concentration to 100 µg/ml. The culture was shaken at 37°C for ~18 hr during which time the culture’s density increased ~5-fold. Then, glycerol was slowly added to a final concentration of 15%. Finally, individual aliquots were snap-frozen in dry ice and ethanol and stored at -80°C.

**Library Enrichments**

All library enrichments were done in 50 ml polypropylene, conical Falcon tubes (Becton Dickinson) with closed lids at 37°C. Experiments were started by thawing an aliquot of transposon library and adding ~1x10^8^ cells to 25 ml media without tobramycin.

For planktonic experiments, tubes were shaken at 250 rpm. For each round, cultures (without tobramycin) were initially grown for 24 hr without tobramycin. Then, 0.3 ml of culture was centrifuged to remove the old media, and the cells were added to 25 ml of fresh media either with or without tobramycin and grown for an additional 24 hr. The next round was started by centrifuging either 0.5 ml of culture from no drug experiments or 1 ml of culture from tobramycin experiments, which grew to a lower density, and suspending the pellets in 25 ml of fresh media without drug. After two rounds, the cultures were centrifuged, the supernatant was discarded, and the cell pellets were frozen at -80°C.

For biofilm enrichments, sterile, plastic slides (Fisherbrand: Cat. #S67112A) were placed in the Falcon tubes, which were not shaken. For each round of enrichment, cultures were grown for 24 hr without tobramycin to allow biofilm formation. Then, the media was slowly drained from the tube through a hole created in the bottom with a hot, blunt needle. The slide, with biofilm attached, was then carefully lowered into 30 ml of fresh media, with or without tobramycin, in a new tube and incubated for another 24 hr. To start the next round, the media was again drained through the bottom of the tube, and the slide was transferred into 30 ml of fresh media without drug in a new tube. After two and a half rounds (i.e., two exposures to tobramycin followed by 24 hr of recovery without drug), the spent media was drained and the slide with attached biofilm was moved to 30 ml fresh media. Then, the biofilm was disrupted through vigorous shaking and vortexing. Cells were pelleted by centrifugation at 2600 g for 15 min, and all but 5 ml of the media was removed. The pellet was suspended in the remaining media, and in order to obtain sufficient material for analysis, the tube was shaken at 37°C until the culture reached late exponential phase. At that point, the culture was centrifuged, the supernatant was removed, and the cell pellet was stored at -80°C.

**Genetic Footprinting**

Genomic DNA was isolated from frozen cell pellets using the QIAamp DNA Mini Kit (Qiagen) following the manufacturer’s directions for Gram-positive and difficult-to-lyse bacteria and eluted into water in the final step. Half of the cells from each enrichment were used as starting material.

DNA manipulations were similar to those described before but were altered to accommodate the high GC content of *P. aerguinosa*. Additionally, a second T7 promoter was included in the transposon construct to allow the use of arrays with probes to only a single DNA strand, and labeling was carried out with biotin instead of Cy dye.

Genomic DNA from each sample was split into thirds and subjected to one of three restriction digestions that produced 5’-CG overhangs: BsaHI/ClaI/BstBI/AclI in NEBuffer 4 with 100 μg/ ml BSA, NarI/HpyCH4IV in NEBuffer 1, or HinP1I in NEBuffer 2. Digestions were incubated at 37°C for 3 hr followed by 20 min (60 min for the NEBuffer 4 combination) at 65°C. Following digestion, DNA was ethanol precipitated and re-suspended in water. Enzymes were purchased from New England Biolabs.

Y-linkers were ligated to the restriction-digested DNA by mixing 600 ng of DNA from each of the BsaHI/ClaI/BstBI/AclI and NarI/HpyCH4IV digestions, 300 ng DNA from the HinP1I digestion, 400 units T4 DNA ligase (New England Biolabs), and 120 pmol Y-linker in 30 µl of 1X ligase buffer. The mixture was allowed to drop slowly from room temperature to 4°C overnight. The sample was cleaned with the QIAquick PCR Purification Kit (Qiagen) and eluted in 30 µl of water at the final step.

Next, the DNA adjacent to both ends of the transposon was PCR-amplified with GC-Rich Taq (Roche) using one primer complementary to the Y-linker and one primer from the end of the transposon. Primer sequences are as follows:

Y-linker primer:

ACTACGCACGCGACGAGACG

Transposon Primers:

CTAGCCGCGGGACCGAGATAGGGTTGAGTG

or

GACGATCCCGCAGTGGCTCTCTATACAAAGTT

Each 50 µl reaction contained 1 µl Taq, 10 µl 5X reaction buffer, 10 µl 5 M GC Resolution Solution, 4 µl dNTP mix (2.5 mM each dATP, dCTP, dGTP, dTTP), 5 µl of 10 µM solution of each primer, and 5 µl ligation product. Cycling conditions were 95°C for 2 min; 30 cycles of 95°C for 30 s, 68°C for 30 s, and 72°C for 3 min; and 72°C for 10 min. Samples from each primer pair were cleaned separately with the Qiaquick PCR Purification Kit (Qiagen) according to the manufacturer’s instructions except that three PE washes were performed. Product was eluted in 30 µl water.

Sequences were further amplified by *in vitro* transcription with the MEGAscript T7 kit (Ambion) according to manufacturer’s directions using 0.5 µg of each PCR reaction (1 µg total) as template. The RNA was purified using the RNeasy Mini Kit (Qiagen).

RNA was reverse-transcribed into biotin-labeled cDNA as follows. First, 5 µg RNA and 3.75 µg random hexamers (GE Healthcare) in a total volume of 5 µl of RNase-free water were heated at 70°C for 10 min and then transferred to ice for at least one minute. Next, 3 µl 5X first strand buffer (Invitrogen), 1.5 µl 0.1 M dithiothreitol, 1 µl water, 1 µl dNTP mix (7.5 mM each dATP, dGTP, dTTP; 2.5 mM dCTP), 2 µl 1 mM biotin-aha-dCTP (Invitrogen), 0.5 µl 40 U/µl RNAsin (Promega), and 1 µl Superscript RT III (Invitrogen) were added. The mixture was incubated at 25°C for 10 min, 46°C for 30 min, and 50°C for 2 hr. The reaction was terminated by heating at 95°C for 5 min and snap cooling on ice. RNA was degraded by adding 0.5 µl of 2 mg/ml RNase A (Invitrogen) and incubating at 37°C for 30 min followed by adding 7.5 µl 0.1 N NaOH and heating at 65°C for 30 min. The mixture was then cooled to room temperature and neutralized with 7.5 µl 0.1 N HCl. The sample was ethanol precipitated and re-supsended in water.

cDNA was fragmented to approximately 50-200 bp using 0.3 U/µg DNase I (Pierce) in 10 mM tris acetate, pH 7.5, 10 mM magnesium acetate, and 50 mM potassium acetate. The mixture was heated at 37°C for 10 min, and then the DNase I was inactivated by heating to 98°C for 10 min.

Five micrograms of fragmented, biotin-labeled cDNA in 45 µl of the DNase I digestion buffer were hybridized to GeneChip *P. aeruginosa* Genome Arrays (Affymetrix) according to the manufacturer’s instructions except that the amount of dimethyl sulfoxide was increased to 10.2 µl per 130 µl of hybridization cocktail. Washing and scanning were done according to the manufacturer’s instructions.

**Analysis of Genetic Footprinting Hybridizations**

To define the set of genes shown in Figure 2A, sum-normalized hybridization scores from each experimental selection were divided by the average, sum-normalized signal from two hybridizations of the unselected library, which accounted for the differential abundance of insertions in the original library. As the sum-normalized values corresponding to no signal varied between the conditions due to differential selection stringencies, values less than 20 were set to 20 (empirically chosen) prior to normalizing by the original abundance. Then, we retained only genes that increased at least two-fold in both replicates of at least one experimental condition compared to the unselected library; we defined this to be the set of “beneficial” insertions. To further select for insertion sites causing differential fitness, the fold change between two different experimental conditions of interest needed to be at least 5-fold. Applying these criteria yielded 586 genes that showed a substantial, condition-specific fitness increase in at least one experimental condition (Figure 2A). Simulations of randomly generated data that preserved the variance present between each pair of biological replicates but gave all samples for each gene the same mean indicate that ~3 genes would be included in the set by chance. Hence, we expect ~3 false positives among the set of 586 genes in Figure 2A. Before clustering the data, the two unselected, library-normalized ratios for each condition were combined so that the value closest to one (smallest effect) was used when the repetitions showed consistent behavior (i.e., both greater than one or both less than one); when one repetition showed a beneficial effect and the other showed a deleterious effect, a value of one (no effect) was used. Data was clustered in Matlab using a K-means algorithm with Pearson’s correlation coefficient as the distance metric. Then, considering the data points from all 8 enrichments (i.e., 4 selections, 2 biological replicates each), those genes whose correlation coefficient with the rest of the cluster was less than 0.6 were removed.

**Competition Assays**

The competitions employed a scaled-down version of the experimental setup used for the enrichments. Competitions took place at 37°C in closed, 2 ml microfuge tubes containing 1 ml of media. To start a competition, roughly equal amounts of overnight cultures of the CFP-labeled reference strain (SAH349) and the YFP-labeled mutant strains were mixed and 10 µl of the mixture was added to a microfuge tube. For planktonic competitions, the tubes were shaken at 250 rpm, and 10 µl of culture was transferred to fresh media every 24 hr. For biofilm competitions, tubes contained a piece of plastic slide and were not shaken; the slide was moved to new media every 24 hr. For both the no drug and tobramycin competitions, cells spent the first 24 hr in media without drug. For the tobramycin competitions, the second 24 hr took place in media with tobramycin. For all competitions, on the third day, a final transfer was made to media without drug, and biofilms were removed from the slides by vigorous shaking and vortexing. In order to obtain sufficient signal and to minimize the contribution of non-viable cells, tubes were shaken at 37°C for 4-8 hr until the cultures reached late exponential phase. Then, cells were pelleted by centrifugation and washed several times in PBS to remove pyoverdine, which is soluble and has excitation and emission spectra similar to CFP . Finally, the pellets were suspended in PBS, moved into black 96-well plates with clear bottoms (Costar), and CFP and YFP signals were read using a SynergyMx platereader (Biotec).

Calculations to determine relative changes in abundance accounted for the initial population sizes, as judged by the absorbance of the original, individual cultures at 600 nm. To account for possible fitness alterations resulting from the tetracycline resistance cassette present in the mutants and differences between the mutant and the reference strain backgrounds, the average result of competitions between 10 strains chosen at random from the U. of Washington collection (SAH076-SAH085) and SAH349 was subtracted from the values reported. SAH084 was the most ‘average’ of the random mutants tested and hence was included as a reference strain in the PA3726 mutant expression study and in the growth rate assays.

Overall, the set of 45 mutants chosen for detailed characterization contained 22 from the ‘Bio TOB Only’ cluster, 2 from the ‘Planktonic TOB Only’ cluster, and from 12 the ‘TOB (Both)’ cluster (Figure 2A). This set mostly included mutants that were at least 3-fold enriched in the Bio-TOB experiment compared to both the Pla-TOB and Bio-ND challenges. To capture insertion events that might be reflected by the signal from a subset of probes for each gene as would be expected for insertions in intergenic regions, additional analysis was carried out at the probe level, which contributed 3 mutants (PA2751, PA3751, and PA4175) to the final list of 45. The remaining mutants either had very high signals following the Bio-TOB enrichment but insufficient increase in population share compared to the unselected library for inclusion in Figure 2A (PA2380 and PA3844), had moderately high signal following the Bio-TOB enrichment and annotations suggesting potential involvement (PA3064, which encodes PelA , and PA5361, which encodes PhoR , or were included as controls (PA5451).

**Growth Curves**

Cultures were grown overnight in M63 media in microfuge tubes and diluted either 1:150 (for growth curves with tobramycin) or 1:100 (for all other assays) into 150 µl media in 96-well plates. Carbenicillin was included when needed for reporter plasmid maintenance. Non-fluorescent assays used clear plates (Costar #3370); fluorescent assays used black plates with clear bottoms (Costar #3637). Mineral oil (100 µl) was layered over each well. The absorbance at 600 nm was read every 15 or 20 min in a SynergyMx plate reader (Biotec). GFP fluorescence (emission 488 nm, excitation: 510 nm) was measured when needed. Strains were assayed at least in duplicate on different days.

Growth curves with 0, 4, and 8 µg/ml tobramycin were generated for the following strains: SAH018 (PA1329), SAH020 (PA0748), SAH027 (*nuoK*), SAH029 (*lasR*), SAH032 (PA2771), SAH041 (*rhlI*), SAH065 (PA0614), SAH084 (MPAO1), SAH108 (PA3726), SAH110 (PA4516), SAH112 (PA1732), SAH113 (*nuoA*), SAH114 (PA2653), SAH116 (PA3048), SAH121 (*mexE*), SAH124 (PA3844), SAH127 (*pscJ*), SAH128 (PA3222), SAH129 (PA3966), SAH130 (PA5207), SAH132 (*wzm*), SAH318 (*rbsB*), SAH320 (*purT*), and SAH328 (PA4175).

**List of GEO Expression Datasets Used for Microarray Expression Meta-Analysis**

The following *P. aeruginosa* expression datasets were downloaded from the Gene Expression OmniBus database (http://www.ncbi.nlm.nih.gov/geo/) and used for microarray expression meta-analysis: GSE2430, GSE4026, GSE6122, GSE6741, GSE7266, GSE7704, GSE7968, GSE8083, GSE8408, GSE8953, GSE9255, GSE9592, GSE9621, GSE9657, GSE9926, GSE9989, GSE9991, GSE10030, GSE10065, GSE10304, GSE10604, GSE11544, GSE12207, and GSE14253.

**Comparative Promoter Activity**

Comparative promoter activity measurements were carried out similar to library enrichment experiments in 50 ml tubes. MPAO1 cells carrying the appropriate *gfp­*-fused promoter construct were grown overnight in M63 and used to start all planktonic and biofilm experiments (~1×10^8^ cells). In all planktonic experiments, tubes were shaken at 250 rpm; for biofilm enrichments, a sterile, plastic slide was provided as the biofilm formation substrate and cultures were not shaken. All the experiments were carried out at 37°C in the presence of carbenicillin. For each round of enrichment, cultures were grown for 24 hr without tobramycin. Then, 1/100th of the old culture (for planktonic experiments) or the slide (for biofilm experiments) was transferred to fresh media either with or without tobramycin and grown for an additional hour. A fraction of cells from the planktonic culture or disrupted biofilm was used to determine the promoter activity (*gfp* fluorescence). The reported value for each case is CFU-normalized. CFUs were determined by plating serial dilutions of the samples.

**Sample Preparation for Expression Analysis**

Overnight cultures were diluted 100-fold into fresh media and shaken at 250 rpm at 37°C until the cultures reached mid-exponential phase (OD_600_ =0.3 on an Ultraspec 3100 pro). Then, 2 ml of culture was added to 4 ml of RNAprotect Bacteria Reagent (Qiagen). The mixture was incubated at room temperature for 5 min and then centrifuged at 5000 g for 10 min. The supernatant was removed and the pellets were stored at -80°C. RNA was isolated using the RNeasy Mini Kit and an initial incubation with 5 mg/ml lysozyme in TE and an on-column DNA digestion. The RNA was converted to cDNA, fragmented, labeled with biotin, and hybridized to Affymetrix GeneChip *P. aeruginosa* Genome Arrays according to the manufacturer’s directions with two exceptions. First, the amount of dimethyl sulfoxide was increased to 10.2 µl per 130 µl of hybridization cocktail. Second, the cDNA synthesis protocol was slightly modified. Briefly, 20 µg RNA and 1.5 µg random hexamers in a total volume of 50 µl were heated at 70°C for 10 min and then cooled on ice. Next, 16 µl of 5X Superscript III 1st strand buffer (Invitrogen), 4 µl of 0.1 M dithiothreitol, 4 µl dNTP mix (10 mM each dATP, dCTP, dGTP, and dTTP), 2 µl SUPERase In (20 U/µl), and 4 µl Superscript III were added. The mixture was incubated at 25°C for 10 min, 37°C for 10 min, 46°C for 10 min, and 50°C for 2 hr. The reaction was terminated by heating at 70°C for 10 min and then held at 4°C. Next, 30 µl of 1 N NaOH were added. After incubating at 65°C for 30 min, the reaction was cooled to room temperature and neutralized with 30 µl 1 N HCl. Finally, the cDNA was purified with the QIAquick PCR Purification Kit (Qiagen).

**References**

1. Liberati NT, Urbach JM, Miyata S, Lee DG, Drenkard E, et al. (2006) An ordered, nonredundant library of Pseudomonas aeruginosa strain PA14 transposon insertion mutants. Proc Natl Acad Sci U S A 103: 2833-2838.

2. Goodman AL, Kulasekara B, Rietsch A, Boyd D, Smith RS, et al. (2004) A signaling network reciprocally regulates genes associated with acute infection and chronic persistence in Pseudomonas aeruginosa. Dev Cell 7: 745-754.

3. Scurlock TR, Miller RV (1979) PaeExo IX: a unique deoxyribonuclease from Pseudomonas aeruginosa active in the presence of EDTA. Nucleic Acids Res 7: 167-177.

4. Girgis HS, Liu Y, Ryu WS, Tavazoie S (2007) A comprehensive genetic characterization of bacterial motility. PLoS Genet 3: 1644-1660.

5. Elliott RP (1958) Some properties of pyoverdine, the water-soluble fluorescent pigment of the pseudomonads. Appl Microbiol 6: 241-246.

6. Kirisits MJ, Prost L, Starkey M, Parsek MR (2005) Characterization of colony morphology variants isolated from Pseudomonas aeruginosa biofilms. Appl Environ Microbiol 71: 4809-4821.

7. Zaborin A, Romanowski K, Gerdes S, Holbrook C, Lepine F, et al. (2009) Red death in Caenorhabditis elegans caused by Pseudomonas aeruginosa PAO1. Proc Natl Acad Sci U S A 106: 6327-6332.
