# Supplementary material for: Fitness Landscape of Antibiotic Tolerance in Pseudomonas aeruginosa Biofilms
Source: PLoS Pathog. 2011 Oct 20;7(10):e1002298. doi: 10.1371/journal.ppat.1002298 (PMC3197603; doi:10.1371/journal.ppat.1002298)
Supplement: Figure S2 — Competitive enrichment data for 45 genes selected for further analysis. Shown is the genome-wide footprinting data for the 45 genes chosen for further analysis. See the ‘Competition Assays' section of Protocol S1 for selection criteria. Genes (rows) were arranged using hierarchical clustering, and the hybridization scores shown for each gene were mean-centered and normalized to a standard-deviation of one. Column labels indicate the experimental condition: Bio-ND and Bio-TOB refer to transposon insertion libraries grown as a biofilms and treated with no drug or tobramycin, respectively, and Pla-ND and Pla-TOB refer to libraries grown planktonically without or with tobramycin. Two biological replicates were performed in each condition, and numbers indicate the repetition number. Yellow (blue) indicates that transposon insertions were beneficial (deleterious) in the experimental condition. (PDF) [file ppat.1002298.s005.pdf]

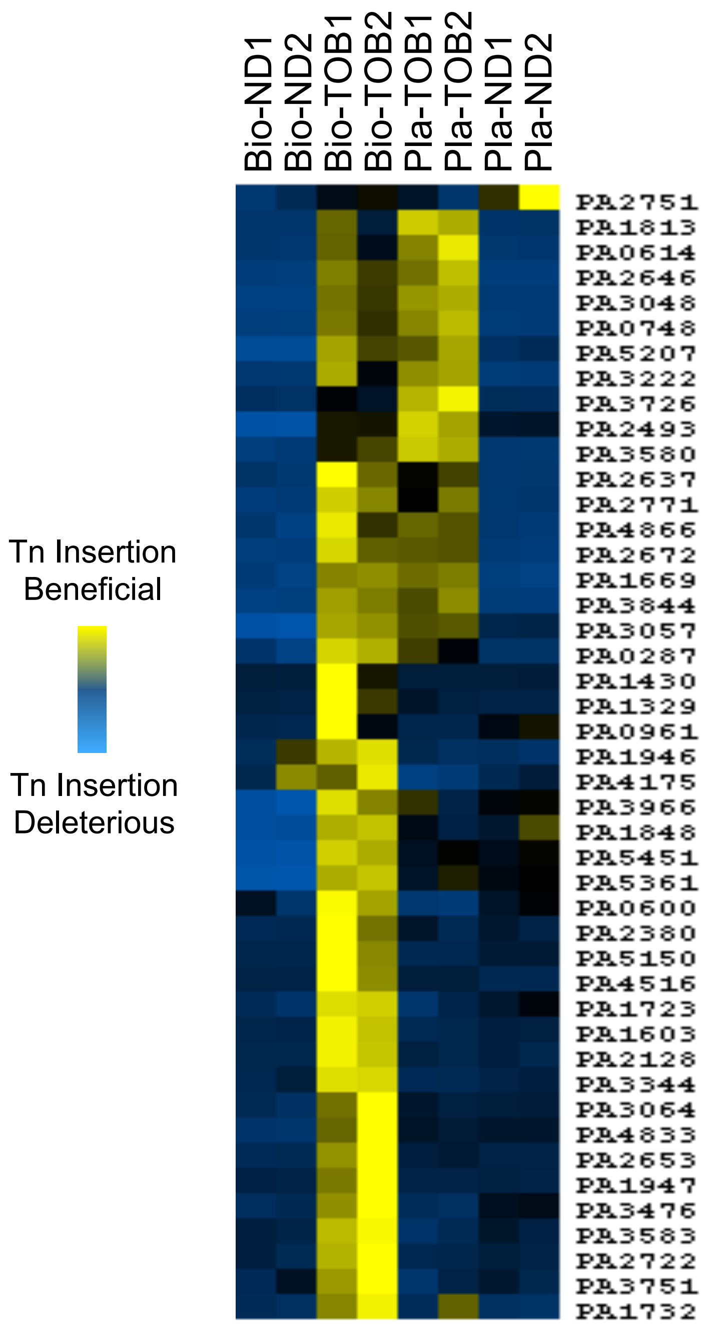
Bio-ND1
Bio-ND2
Bio-TOB1
Bio-TOB2
Pla-TOB1
Pla-TOB2
Pla-ND1
Pla-ND2
Tn Insertion Beneficial
Tn Insertion Deleterious
PA2751
PA1813
PA0614
PA2646
PA3048
PA0748
PA5207
PA3222
PA3726
PA2493
PA3580
PA2637
PA2771
PA4866
PA2672
PA1669
PA3844
PA3057
PA0287
PA1430
PA1329
PA0961
PA1946
PA4175
PA3966
PA1848
PA5451
PA5361
PA0600
PA2380
PA5150
PA4516
PA1723
PA1603
PA2128
PA3344
PA3064
PA4833
PA2653
PA1947
PA3476
PA3583
PA2722
PA3751
PA1732
